# Securing Smart Healthcare Cyber-Physical Systems against Blackhole and Greyhole Attacks Using a Blockchain-Enabled Gini Index Framework

**DOI:** 10.3390/s23239372

**Published:** 2023-11-23

**Authors:** Mannan Javed, Noshina Tariq, Muhammad Ashraf, Farrukh Aslam Khan, Muhammad Asim, Muhammad Imran

**Affiliations:** 1 Department of Avionics Engineering, Air University, Islamabad 44000, Pakistan; 211858@students.au.edu.pk (M.J.); noshina.tariq@mail.au.edu.pk (N.T.); muhammad.ashraf@mail.au.edu.pk (M.A.); 2 Center of Excellence in Information Assurance (CoEIA), King Saud University, Riyadh 11653, Saudi Arabia; 3 Department of Cyber Security, National University of Computer and Emerging Sciences, Islamabad 44000, Pakistan; muhammad.asim@nu.edu.pk; 4 Institute of Innovation, Science, and Sustainability, Federation University, Brisbane, QLD 4000, Australia; m.imran@federation.edu.au

**Keywords:** smart healthcare system, cyber-physical systems, fog, blackhole attacks, greyhole attacks, Gini index, blockchain, trust

## Abstract

The increasing reliance on cyber-physical systems (CPSs) in critical domains such as healthcare, smart grids, and intelligent transportation systems necessitates robust security measures to protect against cyber threats. Among these threats, blackhole and greyhole attacks pose significant risks to the availability and integrity of CPSs. The current detection and mitigation approaches often struggle to accurately differentiate between legitimate and malicious behavior, leading to ineffective protection. This paper introduces Gini-index and blockchain-based Blackhole/Greyhole RPL (GBG-RPL), a novel technique designed for efficient detection and mitigation of blackhole and greyhole attacks in smart health monitoring CPSs. GBG-RPL leverages the analytical prowess of the Gini index and the security advantages of blockchain technology to protect these systems against sophisticated threats. This research not only focuses on identifying anomalous activities but also proposes a resilient framework that ensures the integrity and reliability of the monitored data. GBG-RPL achieves notable improvements as compared to another state-of-the-art technique referred to as BCPS-RPL, including a 7.18% reduction in packet loss ratio, an 11.97% enhancement in residual energy utilization, and a 19.27% decrease in energy consumption. Its security features are also very effective, boasting a 10.65% improvement in attack-detection rate and an 18.88% faster average attack-detection time. GBG-RPL optimizes network management by exhibiting a 21.65% reduction in message overhead and a 28.34% decrease in end-to-end delay, thus showing its potential for enhanced reliability, efficiency, and security.

## 1. Introduction

Cyber-physical systems (CPSs) are a combination of computer and physical technologies that are revolutionizing numerous industries. Modern computing, communication, and control systems are combined with physical processes to facilitate intelligent real-time interactions with the physical world. These systems employ networks of sensors, actuators, and embedded computational devices in order to monitor, evaluate, and control physical processes [1]. Smart healthcare, smart cities, self-driving automobiles, automation in industries, and smart environment monitoring are just a few of the numerous applications of CPS [2]. CPSs enable real-time data collection, analysis, and decision making through the seamless integration of real and virtual environments. CPSs are transforming industries and society by enhancing productivity, safety, and sustainability [3]. CPSs have a tremendous amount of potential for influencing a future in which the physical and digital worlds are closely connected.

Smart healthcare through CPS stands as a transformative force, revolutionizing the landscape of medical care delivery. The importance of integrating CPSs in healthcare lies in their ability to enhance patient outcomes, optimize resource utilization, and streamline the entire healthcare ecosystem [4]. Through interconnected devices, real-time monitoring, and data analytics, smart healthcare systems offer a comprehensive and personalized approach to patient care. Remote patient monitoring, predictive analytics, and smart medical devices contribute to early disease detection, allowing for proactive interventions and personalized treatment plans [5]. The advantages extend beyond individual patient care to the optimization of healthcare operations. CPSs facilitate efficient resource management, reducing costs, minimizing errors, and enhancing overall system resilience. Additionally, the seamless exchange of information among healthcare stakeholders ensures coordinated and timely interventions, improving the quality of care across the continuum [6]. In essence, smart healthcare through CPS empowers healthcare professionals with valuable insights and ensures a patient-centric, data-driven, and interconnected healthcare ecosystem.

An exemplary real-life manifestation of smart healthcare through CPS is evident in the deployment of remote patient monitoring systems [7], such as the “Tele-ICU” programs implemented in various hospitals. These CPSs leverage a network of interconnected medical devices and sensors to monitor patients in real time, even from remote locations. In critical care settings, where timely interventions can be life-saving, Tele-ICU programs utilize smart monitoring devices to continuously track vital signs and other relevant health parameters [8]. The collected data are transmitted securely to a central hub, where healthcare professionals can analyze and respond to emerging trends or anomalies promptly. This not only enables early detection of potential health complications but also facilitates timely adjustments to treatment plans [9]. Importantly, these systems enhance the efficiency of healthcare delivery by reducing the need for constant bedside presence, optimizing resource allocation, and increasing the geographic range of critical care expertise. The successful implementation of Tele-ICU programs shows how smart healthcare CPS can bridge geographical gaps, improve patient outcomes, and redefine smart healthcare [10].

**Figure 1:**
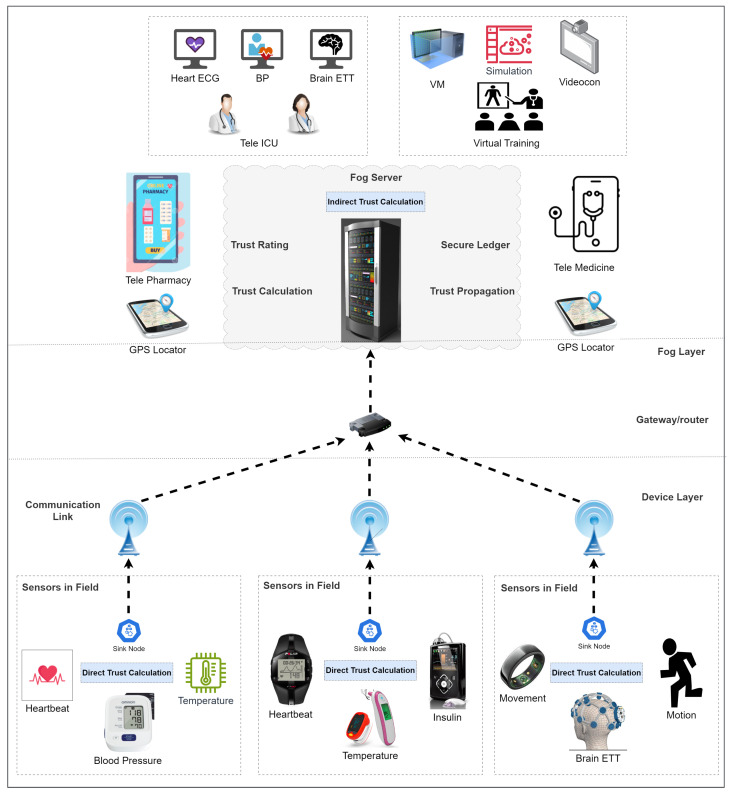
Smart healthcare cyber-physical systems.

Smart healthcare CPS systems are revolutionizing patient care and healthcare delivery. Wearable health trackers such as smart watches and fitness bands have enabled individuals to monitor their activity levels, heart rate, and sleep patterns in real time [11]. Remote patient monitoring devices, including blood pressure monitors and glucose meters, empower patients to manage chronic conditions from the comfort of their homes while providing healthcare professionals with valuable data for proactive interventions [7]. Implantable medical devices, like pacemakers and insulin pumps, have facilitated continuous monitoring and adjustment of physiological functions [12]. Tele-health platforms facilitate virtual consultations, connecting patients with healthcare providers regardless of geographical distances. Smart pill dispensers and inhalers contribute to medication adherence, enhancing treatment efficacy. The pervasive use of IoT-enabled medical equipment and smart health apps further underscores the interconnected nature of these systems [13]. These CPS devices in smart healthcare systems are promoting personalized, efficient, and patient-centric healthcare practices. A generic layout of the smart healthcare CPS is described in Figure 1.

CPSs are open to such attacks because the attackers try to take advantage of the integrated environment. Unauthorized access to a CPS is a serious threat because it opens the door for malicious actors to penetrate the network and impair system performance [14]. Similarly, the injection of fake data can deceive the system’s decision-making procedures and jeopardize its integrity [15]. Additionally, malware, viruses, or ransomware disrupt the regular operation of CPSs [16]. As a result, devastating outcomes, including physical harm, financial losses, and sometimes even fatalities, can result from such attacks. Smart healthcare CPSs are significantly at risk from attacks on CPSs, especially blackhole and greyhole attacks [17]. Organizations can monitor user behavior and establish regular activities by using trust-based protection [18]. By swiftly isolating attackers and taking action, decision makers in a smart healthcare CPS may lessen the impact of security breaches and protect their systems and data [19]. Integrating timely security strategies is crucial to ensuring integrity and confidentiality [20,21,22].

To ensure the security and resilience of a CPS, robust detection and mitigation techniques must be utilized to identify and respond to prospective attacks effectively. Diverse strategies and technologies have been developed to detect and mitigate attacks against CPSs in an effort to protect their essential functions and preserve system integrity [23,24,25,26,27]. CPS security researchers are investigating cutting-edge detection and mitigation strategies, such as deep learning (DL) and artificial intelligence (AI) [27,28], in addition to conventional IDS methods [29]. However, it is crucial to remember that detection alone is insufficient; to respond to identified attacks and lessen their effects on CPSs, appropriate mitigation methods must be in place. AI-based solutions are resource-hungry, and they may drain the limited resources of RPL-based networks [30].

To resolve the above issues, this paper presents an efficient detection method called Gini-index and blockchain-based Blackhole/Greyhole RPL (GBG-RPL) for timely detection and mitigation of blackhole and greyhole attacker nodes while focusing on the characteristics of Low-power Lossy Networks (LLNs). The proposed framework utilizes the Gini index to analyze data distribution, enabling real-time detection of internal attacks. Additionally, blockchain technology ensures data integrity through immutability, smart contracts, and decentralized features, mitigating single points of failure (SPOF) and ensuring scalability. The integration of the Gini index and blockchain strengthens the system’s capacity to identify and isolate malicious nodes, contributing to improved network resilience against potential blackhole and greyhole attacks. The framework ensures the integrity and reliability of healthcare data, building confidence among patients, healthcare professionals, and stakeholders. The GBG-RPL technique provides accurate detection and isolation of the attacker nodes by utilizing the Gini index to calculate the trust values of each node. Furthermore, all trust calculations, along with blockchain implementation, have been shifted to fog nodes at the fog layer, which further contributes to the efficiency of the smart healthcare system. Thus, the application of the proposed framework in smart healthcare systems would add to the security and efficacy of smart healthcare services. In part Abbreviation we defines the abbreviations used in this paper. The salient contributions of this research are as follows:

1. A layered trust framework is proposed to mitigate SPOF and improve the overheads related to messages, energy, and computations in smart healthcare CPSs.
2. A decentralized and scalable framework is presented, enhancing security against blackhole and greyhole attacks using the Gini index.
3. Integration of Gini index and blockchain within the smart healthcare monitoring CPS architecture is proposed to enhance the system’s ability to identify and isolate malicious nodes, improving the network integrity and performance.

The rest of the paper is structured as follows: Section 2 presents the literature review and Section 3 explains the proposed solution. Section 4 describes a case scenario in a smart healthcare CPS, whereas Section 5 presents mechanisms for trust management in the proposed smart healthcare system. Section 6 illustrates the system architecture, and Section 7 describes the CPS architecture in GBG-RPL. Section 8 presents methods for designing, testing, and deploying GBG-RPL in a smart healthcare CPS. Section 9 covers the experimental setup for simulation testing of the proposed framework, while Section 10 describes the results and discussion, followed by Section 11 concluding the paper and presenting the future directions.

## 2. Literature Review

This section provides a detailed literature review and related work on blackhole and greyhole attacks.

### 2.1. Related Work on Trust Mechanisms

A unique method for identifying and mitigating blackhole attacks in 6LoWPAN RPL-based wireless sensor networks (WSNs) is presented by Sharma et al. [31], which is referred to as Blackhole detection in RPL-based CPS (BCPS-RPL) in this paper. By preventing malicious nodes from interfering with network communication, the proposed method improves the security of CPS. A lightweight trust-enabled routing technique has been proposed by Arshad et al. [32] to reduce the impact of Sybil attacks in RPL-based IoT networks. The suggested method efficiently detects and prevents Sybil attacks, and hence, enhances the security and dependability of IoT networks. A taxonomy of several network attacks on CPSs, including denial of service (DoS), data manipulation, and injection attacks, has been provided by Cao et al. [33]. Groves and Pu [34] suggest utilizing the Gini index to identify and isolate Sybil attacks in IoT.

Methods for detecting and preventing greyhole attacks, such as trust-based procedures and routing protocols, have been elaborated in Chinnaraju and Nithyanandam [35]. The study explains the problems that may arise from greyhole attacks and suggests ways to fix them. An exhaustive taxonomy of attacks, including their characteristics, impacts, and detection/prevention methods, is presented by Savoudsou et al. [36]. To detect and prevent attacks in RPL-based networks, Garcia et al. [37] describe a unique IDS architecture that integrates different anomaly-detection approaches. The contribution is a workable IDS solution modified to fit the specific features and needs of IoT networks based on RPL. To improve the safety of IoT networks, Hashemi and Aliee [38] present a novel trust model developed for the IoT. A new method for localization is presented by Kaliyar et al. [39] that includes features for the early detection of Sybil and wormhole attacks in IoT networks. Using RPL control messages, Bang et al. [40] provide a complete overview of various routing attacks and countermeasures. Sybil attacks in WSNs with a cluster topology are detected using a lightweight trust-based framework given by Sujatha et al. [41].

A behavioral intrusion-detection framework for WSNs is proposed by Smith et al. [42]. The prospective applications and benefits of various technologies have been highlighted by Sharma and Verma [43]. Sharma et al. [44] carefully examine multiple attack vectors across multiple layers of the IoT architecture, propose mitigation strategies, and highlight research gaps. In order to identify and neutralize potential security risks in sensor networks, Gamec et al. [45] analyze the actions of nodes at the device layer. Sanders and Yau [46] present a strategy that combines neighbor monitoring with trust-based routing to identify and isolate blackhole attacks. Kale et al. [47] offer a novel strategy that examines network traffic patterns and behavior to find probable blackhole nodes. In order to identify and remove blackhole nodes from the network, Saputra et al. [48] develop an enhanced approach that examines the behavior of nearby nodes. An innovative method presented by Wagle et al. [49] optimizes the energy usage of security measures while assuring efficient threat detection and mitigation.

Makkar et al. [50] present the FedLearnSP framework, which enables model training without disclosing personal information. An innovative fuzzy logic-based intrusion-detection system is presented by Ghosh et al. [51] as their contribution. The categorization and classification of different attacks according to how they affect the CPS’s sensors, actuators, and communication channels are among the contributions made by Dixit et al. [52]. The taxonomy offers a methodical framework for comprehending and evaluating various attack types in CPSs. In their study on network congestion in WSNs, Pandey and Kushwaha [53] analyze the effects of various security threats, such as blackhole, wormhole, and flooding attacks. Chennam et al. [54] address the particular security difficulties CPSs face and offer feasible solutions to lessen the threats found.

Saeed et al. [55] describe a novel method that makes use of the ERT algorithm to identify and treat WSN problems precisely. Alvarez et al. [56] present a unique method for locating and neutralizing hostile nodes that interfere with network connectivity by using heartbeat messages between the member nodes. Pasikhani et al. [57] conduct a thorough literature review, identifying several IDS strategies, their methodologies, and performance in the context of 6LoWPAN networks. A comprehensive comparison of the papers reviewed during the research based on trust mechanisms is given in Table 1.

### 2.2. Related Work on Blockchain Security

By using blockchain technology to store trust data securely and impenetrably, Tariq et al. [17] reduce the drawbacks of current trust models. The suggested technique delivers better internal attack-detection accuracy and security. A strong and trustworthy model is provided by Sivaganesan [60], which focuses on data-driven techniques to detect and mitigate attacks. Guo et al. [62] have analyzed the uses, advantages, and difficulties of blockchain in different fields. Blockchain in IoT environments has been analyzed along with problems of incorporating it into trust-management systems by Liu et al. [63]. For the purpose of determining the advantages, difficulties, and future applications of combining blockchain with cloud computing, Gong and Navimipour [64] thoroughly review and synthesize the existing research. In addition to highlighting the security issues in fog computing, the study by Alzoubi et al. [61] presents possible applications of blockchain technology to improve security and privacy. A detailed overview of several blockchain-based security solutions for the IoT is provided in the paper by Khan et al. [65]. A comprehensive comparison of the papers reviewed during the research based upon blockchain security is given in Table 2.

## 3. Proposed Solution

The main features of the proposed Gini index and blockchain based solution are listed as follows:

1. Integration of the Gini index and blockchain for attack detection and mitigation: When applied to CPS security, combining the Gini index and blockchain is an attractive way to improve detection and mitigate attacks. The idea behind integrating these two technologies is that they can enhance CPS security due to overlapping functionalities and synergies [66,67]. The Gini index is used to detect weak spots in a CPS [68] by keeping the focus on resource disproportions. Blockchain technology, on the other hand, has the advantages of being immutable, transparent, and decentralized, all of which strengthen CPS security [69]. Blockchain technology uses SHA-256 (Secure Hash Algorithm 256-bit) as the hashing algorithm. The hash function SHA-256 is well-known for its safety and collision resilience [70]. It generates an output with a fixed length of 256 bits. It is essential to maintaining the immutability and security of data in the blockchain since it creates a distinct hash for every block according to its contents. Blockchain technology combined with the Gini index offers a more reliable and efficient cybersecurity mechanism [67,71].
2. Enhancing CPS security and benefits/synergies of integration: There are multiple methods in which the integration of blockchain with the Gini logic could enhance CPS security. Initially, although blockchain technology safeguards the Gini data’s integrity and immutability, it can also be employed to identify resource disparities and possible attacks [68,69]. Secondly, the openness and decentralized nature of blockchain allow all CPS network users to access and validate the Gini index data, encouraging cooperation and group security initiatives [69]. Additionally, the integration makes it possible for authorized users to safely share and disseminate the Gini index data, enabling real-time monitoring and defense against potential threats [71].
3. Resource consumption at fog layer: All calculations and associated computational load have been moved from the device layer to the fog layer. As a result, the computational load on resource-constrained device layer nodes is reduced, and it is instead distributed to a high-performance fog server, resulting in a decrease in energy usage, message overhead, and end-to-end delay.

### 3.1. Assumptions

The following assumptions are made for the proposed methodology:

1. Initially, all network nodes are trustworthy and contain no malicious nodes.
2. A root node, also known as the LLN Border Router (LBR), is a resourceful computational device and is assumed to be trustworthy during the CPS’s network life.
3. Each device registers with the root node using a special identification number.
4. Other than the root, devices may or may not be mobile; the root will stay static.
5. The communication channel is secure.
6. The attacker node is not intelligent.

### 3.2. Gini Index-Based Trust Model

The Gini index describes the degree of income dispersion across the whole income spectrum by integrating specific share data into a single statistic [72]. The Gini coefficient ranges from 0 to 1, with 0 denoting perfect equality and 1 denoting perfect inequality. It measures the difference between the observed cumulative income distribution, or Lorenz curve, and the idealized case of completely equitable income distribution. Gini impurity, another name for the Gini index, is used to measure the likelihood or severity of misclassification when a variable is chosen at random. When all the elements fall into a single class, a state of purity results, and the idea of “impurity” is introduced.

**Figure 2:**
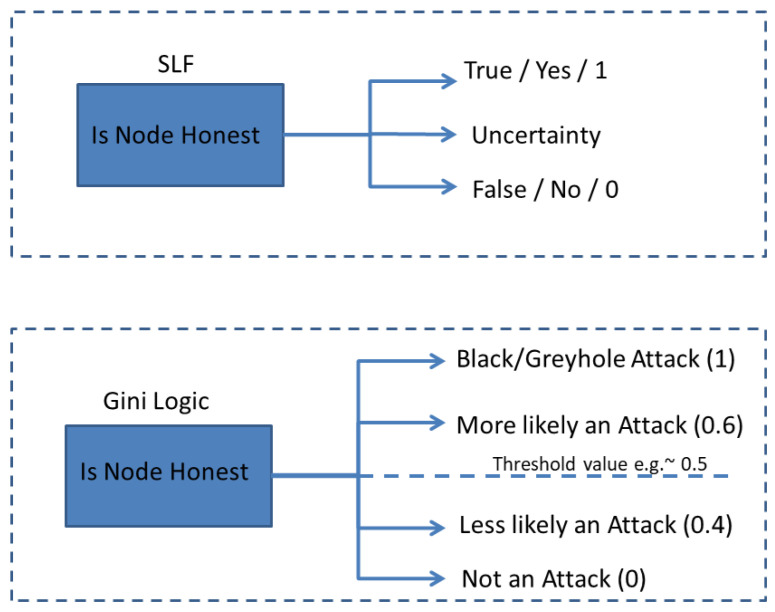
Trust thresholds for Gini index in CPS.

The core idea of the proposed Gini countermeasure is to exploit the statistical properties of entities for the detection and mitigation of blackhole and greyhole attacks. The Gini countermeasure, in particular, evaluates the variation in received DAO messages using the Gini index-based theory, allowing the identification of probable blackhole or greyhole attacks, as depicted in Figure 2. The Gini countermeasure launches the appropriate mitigation steps to lessen the impact of such attacks as soon as they are discovered. The generic mathematical notation of the Gini index for attack detection in a CPS is given below:

1. The Gini index’s degree ranges from 0 to 1.
2. “0” indicates that there is only one class (pure) or that all elements fall under that class.
3. The number “1” indicates that the elements are dispersed at random (impure) throughout the classes.
4. An equal distribution of elements into some classes is indicated by a Gini index value of 0.5.

Let *I* be the Gini index, which represents the inequality of flow distribution in the CPS network, as shown in the Equation (1).

(1) $$I=1-\sum_{i=1}^{n}{\left(a_{i}\right)}^{2}$$

where $a_{i}$ denotes the proportion of flow *i* in a network.

The Gini index idea can be used to evaluate resource allocation and spot potential weaknesses in the context of CPS security [73]. Computational power, bandwidth, and storage are among the resources that are distributed among different system components and entities in CPS. However, an uneven distribution of resources might provide attackers access to vulnerable points, jeopardizing the system’s overall security and functionality. The Gini index becomes important in this situation. By computing the ratio between the cumulative differences in resource allocation and the total allocation, the Gini index gives a quantitative indicator of resource distribution. A higher Gini index value denotes a system with more resource inequality or imbalance. Researchers and practitioners in CPS security can learn more about resource distribution trends and spot potential weak spots by using the Gini index.

The capacity of the Gini index to identify resource imbalances that potential attackers can exploit makes it relevant to CPS security. Security teams can identify and investigate resource allocation abnormalities that could be signs of attacks or unauthorized resource use by tracking the Gini index over time. In order to discover weaknesses and improve the overall security posture of CPS, the Gini index functions as a metric that supplements conventional security measures. The Gini index can also be used to increase security measures and give priority to resource allocation in vulnerable areas [74]. The Gini index assists in reducing the danger of blackhole and greyhole attacks, in which resources are fraudulently devoured or purposely diverted, by correcting resource imbalances and ensuring a more equitable distribution. Given that it provides a quantitative evaluation of resource allocation and risk inside the system, the Gini index notion is extremely pertinent to CPS security. Security professionals can identify potential attack vectors, understand resource imbalances, and prioritize security solutions by using the Gini index [75]. The use of the Gini index improves CPS security’s overall resiliency and efficacy, protecting and preserving the integrity of these intricate and linked systems.

Despite being a relatively recent concept, the application of the Gini index in CPS security has shown promise in related disciplines. The Gini index, for instance, has been used to examine how energy consumption is distributed among sensor nodes in WSNs. The index assists in identifying nodes that consume excessive amounts of energy, highlighting potential weaknesses that attackers could exploit or places where energy-saving techniques could be used [76]. The Gini index has been used in cloud computing systems to evaluate the equity of resource distribution among virtual machines, improving load balancing and system performance [77]. There are a number of possible advantages to using the Gini index in CPS security. Firstly, it gives security professionals a quantifiable measure of resource allocation so that they can identify and prioritize areas that need attention and changes in resource allocation [78]. Secondly, it assists in the identification of crucial parts or subsystems that would require stronger security precautions because of resource concentration. Thirdly, the Gini index helps to spot potential attack vectors and weak spots that bad actors may exploit. [79]. Security solutions can be adapted to safeguard the most important and vulnerable parts of the CPS by evaluating resource allocation patterns. However, there are restrictions to take into account when using the Gini index for CPS attack detection. The Gini index does not offer information about particular attack kinds or methodologies because it primarily concentrates on resource distribution [80]. Furthermore, the accuracy and dependability of resource allocation data have a direct impact on the Gini index’s validity and effectiveness as an attack-detection tool [81].

### 3.3. Trust Calculation in Proposed Methodology

Trust calculation methodologies in CPSs are instrumental in evaluating the trustworthiness of entities within the system. These methodologies employ trust models, algorithms, and selected metrics to assess the behavior, reputation, and history of entities. By considering factors such as past performance, adherence to security protocols, successful task execution, feedback, and reputation, trust values are computed for each entity. These values enable informed decision making, resource allocation, and detection of malicious behavior. Trust calculation methodologies in CPS are adaptive, ensuring accurate and up-to-date trust assessments. A list of symbols used in mathematical notations is given in Table 3. Algorithm 1 represents the detection of malicious nodes in a smart healthcare CPS.
**Algorithm 1:** Malicious node detection.
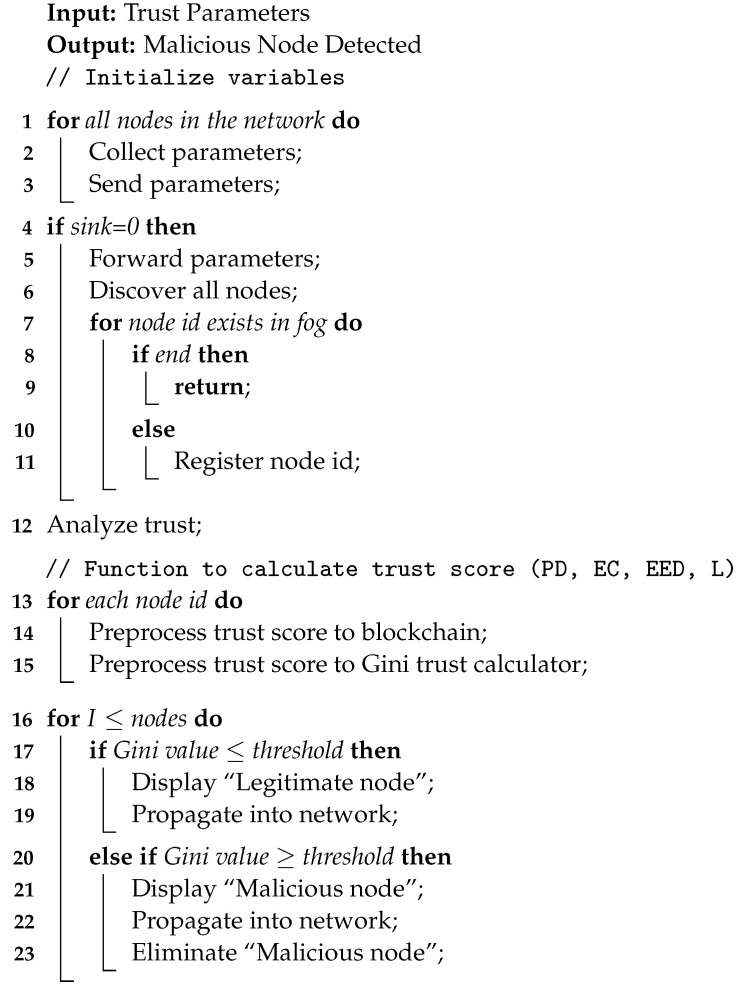

1. Direct Trust Calculation
  Monitoring nodes at the device layer of the CPS is referred to as direct trust calculation. Monitoring nodes at the device layer is performed by resource-constrained devices by continuously tracking and examining the actions and interactions of other nodes within the CPS. The real-time data gathered by these nodes include trust parameters such as the packet drop rate, energy usage, end-to-end delay, and message overhead. Monitoring nodes use trust models and algorithms as part of the direct trust calculation process to assess the reliability and behavior of CPS entities.
  In a CPS, direct trust computation at the device layer offers a number of benefits. First of all, it offers real-time trust evaluation, allowing for the quick detection of malicious or untrustworthy system elements. Second, direct trust computation gives a thorough evaluation of entity trustworthiness by taking into account trust factors such as the packet loss rate, energy consumption, end-to-end delay, and message overhead. Direct trust calculation at the device layer has its challenges. The precision and scalability of trust computations may be impacted by the monitoring nodes’ constrained memory and computational capacity. In order to avoid unauthorized access or manipulation of trust values, it is also essential to ensure the security and privacy of trust data. To address this issue, the proposed methodology incorporates the device layer nodes to gather only the trust parameters and send them to the fog node through a sink node for data aggregation and trust calculation. Once the overall trust calculations are computed at the fog layer, the same are stored in the global trust list and forwarded to all member nodes for subsequent actions. A general mathematical notation for calculating direct trust using the above trust parameters is depicted in Table 4, where EC stands for the energy used, L for the latency, EED for the end-to-end delay, and PDR for the number of dropped packets.
2. Indirect Trust Calculation for Blackhole
  Several features can be taken into account when utilizing the Gini index to find a blackhole node in a CPS. Here are several distinctive characteristics of a blackhole node:
  (a) Deviation in Gini index: The Gini index gauges how uneven or unequal the CPS network’s flow characteristics are. The Gini index values significantly differ when a blackhole node drops packets.
  (b) Packet loss: Incoming packets are purposefully dropped by blackhole nodes, which results in a high packet loss rate.
  (c) Latency: Blackhole nodes have the potential to cause large packet transport or response time delays. Blackhole node anomalous delays can be found by keeping an eye on the communication latency between nodes.
  (d) Energy consumption: Blackhole nodes have higher energy consumption than regular nodes due to packets being dropped.
  (e) Traffic distribution: Blackhole nodes can be found by examining the traffic distribution patterns and locating nodes with unusual or inconsistent traffic distribution.
  In addition to the Gini score, blackhole nodes in CPS networks can be quickly identified by taking into account these properties. These metrics are tracked, analyzed, and compared across the nodes to help find those that behave strangely or might be blackhole nodes. The Gini index’s features for detecting a blackhole node in a CPS can be expressed mathematically as follows:
  Let $G_{m}$ represent the Gini index value of node *m*, $P_{m}$ denote the packet loss rate of node *m*, $L_{m}$ stand for the latency of node *m*, and $EC_{m}$ signify the energy consumption of node *m*. Additionally, let *G* be the set of Gini index values for all nodes in the CPS network, $\bar{G}$ denote the average Gini index value of all nodes in the CPS network, $\theta_{P}$ represent the predefined threshold for packet loss rate, $\theta_{L}$ be the predefined threshold for latency, and $\theta_{E}C$ signify the predefined threshold for energy consumption. These notations play a pivotal role in analyzing the behavior and performance of the CPS network. The characteristics to detect a blackhole node are as follows:
  (a) Deviation in Gini index: The Gini index deviation for node *m* can be defined as shown in Equation (2):
    (2) $$Gini\_Deviation_{m}=\left|G_{m}-\bar{G}\right|$$
  (b) Packet loss: The condition to detect potential blackhole nodes based on packet loss can be expressed as described in Equation (3):
    (3) $$P_{m}>\theta_{P}$$
  (c) Latency: The condition to detect potential blackhole nodes based on latency can be expressed as described in Equation (4):
    (4) $$L_{m}>\theta_{L}$$
  (d) Throughput: The condition to detect potential blackhole nodes based on throughput can be expressed as described in Equation (5):
    (5) $$EC_{m}<\theta_{EC}$$
  (e) Traffic distribution: By analyzing the traffic distribution patterns and identifying nodes with abnormal or inconsistent traffic distribution, blackhole nodes in a CPS network are detected and subsequently eliminated.
  The pseudo-code for the detection of blackhole nodes using the Gini index is described in Algorithm 2. The time complexity of the algorithm is O(n), while the space complexity is also O(nlogn).
**Algorithm 2:** Detection of blackhole nodes.

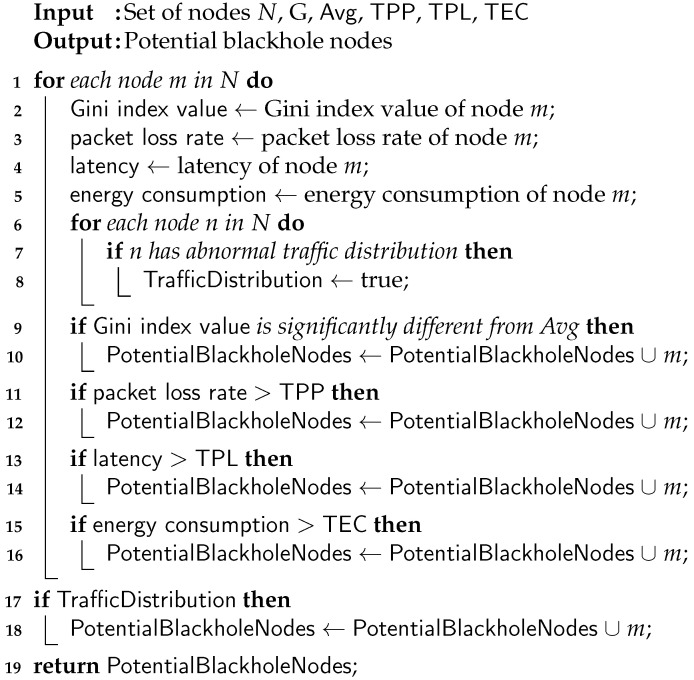

3. Indirect Trust Calculation for Greyhole
  To identify a greyhole node in a CPS, several factors that distinguish anomalous behavior displayed by nodes and indicate the presence of a greyhole node can be taken into account. The following is a list of greyhole node characteristics:
  (a) Gini index deviation: Each node’s Gini index in the CPS network is determined based on the flow characteristics of packet loss, delay, and throughput. Nodes with disproportionately high Gini index values might be greyhole nodes.
  (b) Selective packet dropping: By keeping an eye on a node’s packet-forwarding activity, greyhole nodes can be identified.
  (c) Latency: Greyhole behavior can be detected by tracking the node latency and finding nodes with a higher latency than anticipated.
  (d) Energy consumption: Greyhole nodes can be identified by tracking the energy consumption and spotting those with noticeably low residual energy values.
  (e) Throughput: In comparison to other nodes, greyhole nodes may manipulate or restrict the flow of data, resulting in reduced throughput.
  Greyhole nodes in the CPS can be found by taking into account the criteria mentioned above and the Gini index analysis. The mathematical model below shows how the Gini index can be used to find greyhole nodes in a CPS. Let the characteristics of node *m* at time *T* describe several parameters. The Gini index value is denoted by $G_{m}\left(T\right)$, representing the data distribution. The packet loss rate is indicated by $P_{m}\left(T\right)$, reflecting the rate of lost packets. The latency is represented by $L_{m}\left(T\right)$, capturing the data transmission delay. The energy consumption is denoted by $EC_{m}\left(T\right)$, signifying the power usage. The throughput is indicated by $Th_{m}\left(T\right)$, representing the data transfer rate. θ represents the threshold value for a particular metric and $\bar{G}$ represents the average value of the Gini index. These parameter values are essential for evaluating and managing the performance of node *m* within the CPS network. The characteristics to detect a greyhole node are:
  (a) Gini index deviation: The Gini index deviation for node *m* at time *t* can be defined as described in Equation (6):
    (6) $$Gini\_Deviation_{m}\left(T\right)=|G_{m}\left(T\right)-\bar{G(T})|$$
  (b) Packet loss: The condition to detect potential greyhole nodes based on packet loss can be expressed as described in Equation (7):
    (7) $$P_{m}\left(T\right)>\theta_{P}$$
  (c) Latency: The condition to detect potential greyhole nodes based on latency can be expressed as described in Equation (8):
    (8) $$L_{m}\left(T\right)>\theta_{L}$$
  (d) Energy consumption: The condition to detect potential greyhole nodes based on energy consumption can be expressed as described in Equation (9):
    (9) $$EC_{m}\left(T\right)>\theta_{E}C$$
  (e) Throughput: The condition to detect potential greyhole nodes based on throughput can be expressed as described in Equation (10):
    (10) $$Th_{m}\left(T\right)<\theta_{T}h$$
  The Gini index deviation captures the deviation of a node’s Gini index from the average. At the same time, the packet loss, latency, and throughput characteristics help identify nodes with abnormal behavior in terms of packet loss rate, latency, and data transfer rates, respectively. The pseudo-code for the detection of greyhole nodes using the Gini index is depicted in Algorithm 3. The time complexity of the algorithm is O(n), while the space complexity is also O(nlogn).
4. Trust Update
  A key component of assuring the network’s dependability and security in CPS networks is trust updating. Based on the behaviors and interactions within the network, individual nodes’ given trust ratings are evaluated and updated. There are two basic ways trust updates may occur: routine/periodic updates and reactive updates brought on by modifications in node behavior.
  (a) Routine: Routine trust updating is carried out at predetermined intervals, usually as part of a routine maintenance operation. This method ensures that trust values are always up-to-date and represent the nodes’ current behavior. Network managers may identify potential deviations or anomalies in node activity by periodically analyzing their trustworthiness.
  (b) Reactive: When a node’s behavior changes significantly or displays questionable behavior, reactive trust updating takes place. These adjustments may take the form of abrupt increases in data loss, unforeseen communication delays, or departures from established behavioral norms. When these anomalies are found, a reactive action is taken to adjust the node in question’s trust value.
  For a CPS network to remain trustworthy, both routine and reactive trust-update measures are essential. The routine updates offer a methodical and proactive way to monitor the network, ensuring that trust values are consistently evaluated and modified. Reactive updates, on the other hand, offer a quick way to respond to any abrupt or unexpected changes in node behavior that might point to a security risk. Combining the two strategies enables CPS networks to efficiently respond to dynamic changes in node behavior.

**Algorithm 3:** Detection of greyhole node.

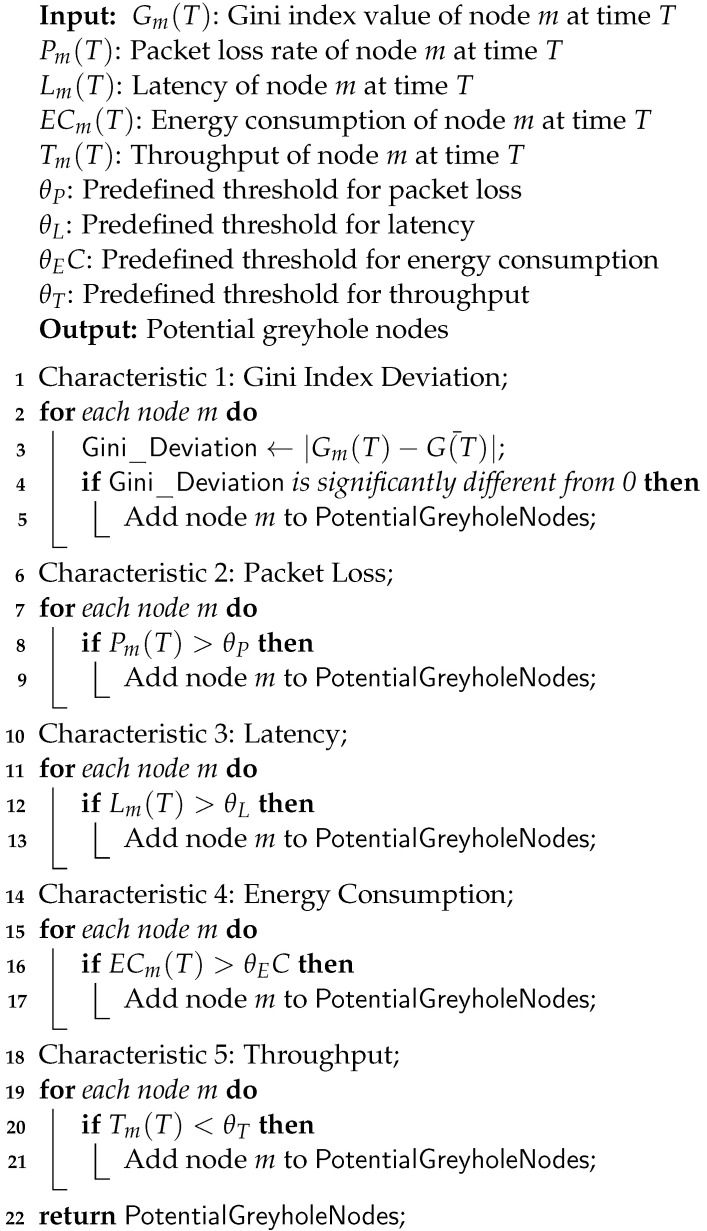




### 3.4. Smart Contracts for Node Registration

In the node-registration process, enhancing security and trust management is made possible by the use of blockchain technology at fog nodes in the fog layer of a CPS. However, implementing blockchain technology and maintaining a global trust list requires resourceful nodes that can be installed at the fog layer. Fog servers can create a safe and automated procedure for adding additional nodes to the CPS network by using smart contracts. In such a scenario, the registration procedure is governed by the smart contract, which serves as a predetermined set of guidelines and requirements. The smart contract receives information from the new node when it tries to join the network, including the node’s identification and any associated metadata. The smart contract then verifies the supplied data using predefined criteria. When the validation is successful, the smart contract creates a blockchain transaction that includes the new node’s identification and any pertinent information. Algorithm 4 represents the functional description of the smart contract module to execute a new transaction and update the blockchain-based revised global trust list (BRGTL). The time complexity of the algorithm is O(1), while the space complexity is O(n).
**Algorithm 4:** Smart contract algorithm.
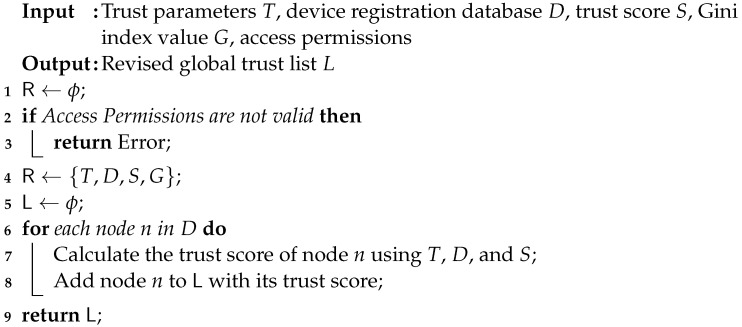


**Figure 3:**
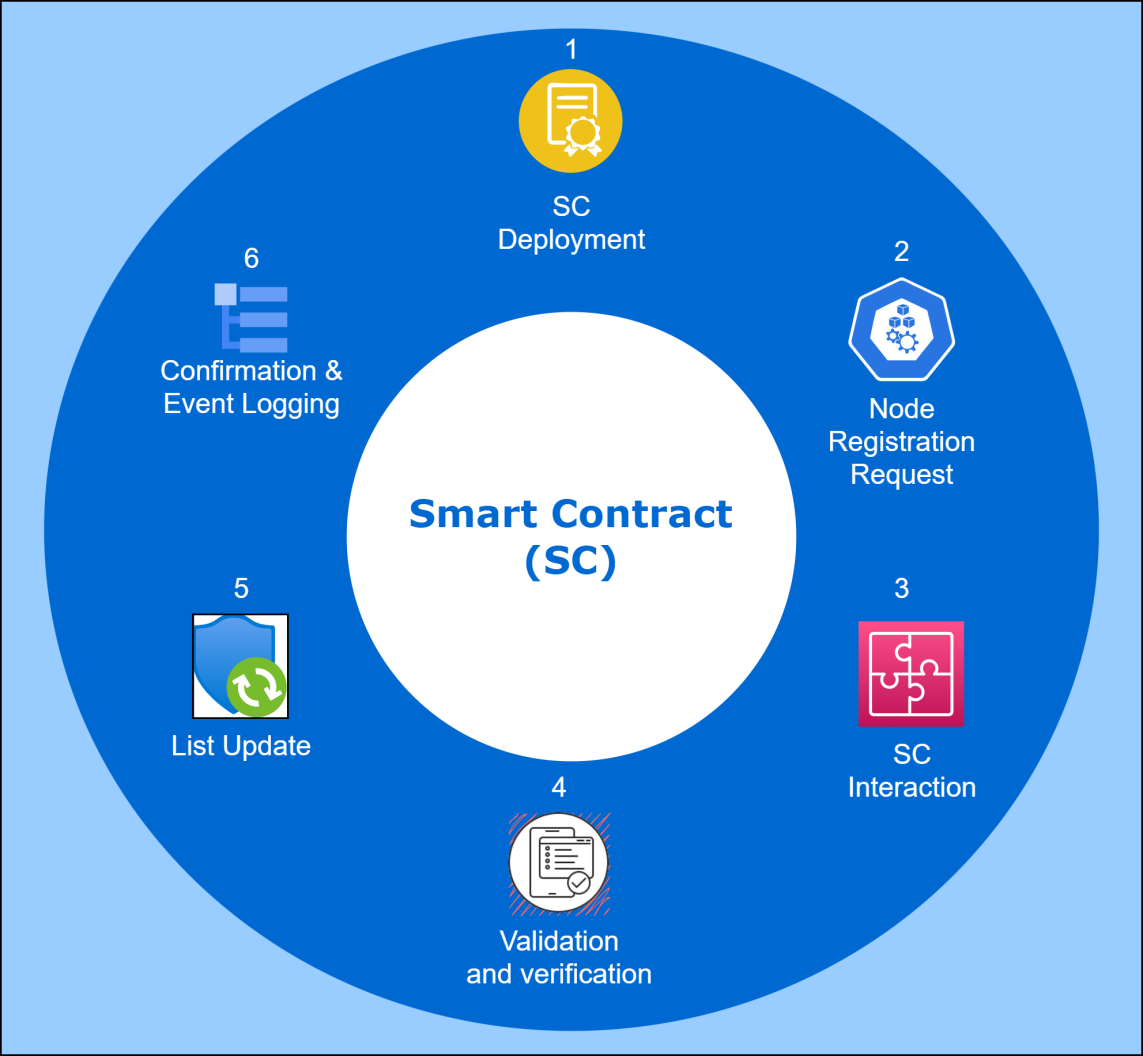
Stepwise flow of smart contract Working at fog layer.

There are a number of security advantages to node registration utilizing smart contracts. First, because the procedure is automated, there is less chance of human error. By confirming the legality of the joining nodes, the smart contract’s validation procedure adds an extra degree of protection. Second, the blockchain’s transparency enables other fog nodes of the same CPS network to audit and validate the registration process. In order to confirm that the registration procedure complies with the established norms and circumstances, they can independently review the transactions that have been recorded on the blockchain. Below is a step-by-step explanation of how smart contracts are implemented at the fog layer, and Figure 3 shows the same.

1. Smart contract deployment: The fog server compiles the smart contract code and then deploys it to the blockchain.
2. Node registration request: A registration request is sent to the fog server by a new node when it wants to join the network. The request contains the data needed for the registration procedure, such as the node’s identification and associated metadata.
3. Smart contract interaction: By calling functions or methods specified in the deployed smart contract’s code, the fog server communicates with it.
4. Validation and verification: After receiving the registration request, the smart contract runs validation and verification tests. The node’s identification is validated by the smart contract, which then confirms its veracity.
5. Updating the registration list: The smart contract updates the node registration list if the registration request is validated successfully. The updated registration information is included in a new transaction that the smart contract makes on the blockchain network.
6. Confirmation and event logging: When the smart contract successfully changes the registration list, it notifies the fog server by sending a confirmation response. The confirmation or event is recorded by the fog server.

### 3.5. Blockchain-Based Gini Index Framework in Smart Healthcare CPS

The proposed framework analyzes the distribution of data using the Gini index. It establishes a baseline of normal data distribution, detecting internal attacks in real time. The adjustment of thresholds based on the Gini index analysis ensures adaptability to evolving system behavior, enhancing the framework’s ability to identify and respond to potential threats. In addition, blockchain technology is integrated to ensure data integrity [17]. The immutability of records on the blockchain creates a tamper-resistant ledger for all transactions. Smart contracts enforce access controls and record transactions, contributing to enhanced data integrity and security [82]. The decentralized nature of blockchain mitigates the risk of SPOF and ensures scalability. The framework is designed to scale effectively, maintaining its security features even in larger and more complex network environments. Therefore, the integration of the Gini index and blockchain enhances the system’s ability to identify and isolate malicious nodes, which is crucial for maintaining network integrity. The combined features of the Gini index and blockchain contribute to improved network resilience, ensuring that the system can withstand and recover from potential blackhole and greyhole attacks effectively.

### 3.6. Deployment Models for Proposed Framework

Various deployment models can be used for the implementation of the proposed framework (GBG-RPL) in the smart healthcare CPS. Each deployment model has its own set of advantages and disadvantages, as tabulated in Table 5.

## 4. A Case Scenario in Smart Healthcare CPS

A hypothetical scenario is described below to illustrate how the proposed framework prevents malicious actors from exploiting vulnerabilities in a smart healthcare CPS.

### Preventing Unauthorized Access to Patient Records

1. Trust parameter vollection: Child nodes within the device layer, such as wearable health trackers and medical sensors, continuously collect trust parameters. These parameters include the packet loss ratio, energy consumption, end-to-end delay, and packet forwarding behavior.
2. Forwarding to fog layer: The collected trust parameters are forwarded to the fog layer through a designated sink or root node. The root nodes forward all received trust parameters to the fog servers for processing and analysis at the fog layer.
3. Device registration: The fog servers register the device if it is not already included in the device-acquirer function and allow the requesting device to become part of the smart healthcare CPS.
4. Gini index calculation: Utilizing the Gini index, the fog layer assesses the distribution of trust parameters across the smart healthcare CPS. Anomalies, such as unexpected spikes in device activity, trigger alerts.
5. Trust updating and blockchain integration: Based on the Gini index analysis, the trust values of nodes are updated in the BRGTL. The updated trust values, along with the trust parameters, are stored in the BRGTL smart contract in the blockchain.
6. Maintaining BRGTL on blockchain: The BRGTL smart contract securely maintains an immutable record of the updated trust values and is accessible to all fog servers. It provides a historical reference for trust assessments and ensures tamper-resistant record keeping.
7. Communication with device layer: The fog layer communicates with the device layer, specifically sharing the updated trust values stored in the BRGTL smart contract. This communication enables devices to be informed of changes in trust assessments.
8. Preventing unauthorized access: Suppose a malicious actor attempts to gain unauthorized access to patient records by exploiting vulnerabilities in a compromised device. The real-time trust assessment, Gini index analysis, and BRGTL record keeping collectively identify this anomalous behavior and isolate the malicious attacker node.
9. Blockchain Security and Immutability: The blockchain’s decentralized and tamper-resistant nature prevents the malicious actor from tampering with THE trust values stored in the BRGTL. Any attempt to manipulate the system is recorded in the Blockchain, maintaining the integrity of the trust assessment.

In the above scenario, the GBG-RPL framework, with its blockchain-enabled Gini index components, successfully prevents malicious actors from exploiting vulnerabilities in a smart healthcare CPS. It establishes a secure, real-time, and centralized system for trust management, ensuring the integrity and confidentiality of patient records.

## 5. Techniques in the Proposed Smart Healthcare CPS for Trust Management

Trust management is essential to guarantee the security and integrity of the proposed smart healthcare CPS. The global trust list (BRGTL), which is based on blockchain, and the Gini index are included in the composition of trust-management mechanisms. Below is a description of these methods’ specifics and how they work together to thwart blackhole and greyhole attacks:

1. Utilization of Gini Index for Trust Assessment
  The distribution of trust parameters gathered from the device layer is subjected to the Gini index in the context of the smart healthcare CPS [34]. Based on the gathered trust parameters, the Gini index is computed, offering insights into how the actions and behaviors of nodes are distributed throughout the network. Unanticipated trends or deviations in the behavior of the devices within the smart healthcare CPS are indicated by irregularities in the Gini index. Alerts are set off by abrupt spikes or notable deviations, which indicate possible security risks [73]. Trust is evaluated in real time by the GBG-RPL. The GBG-RPL adapts, according to the trust parameter changes, making sure the system can react quickly to new security threats.
2. Blockchain for Secure Storage
  A blockchain smart contract forms the basis of the revised blockchain-based global trust list (BRGTL) of the proposed framework. The device parameters, trust values, and trust parameters are all safely stored and kept up-to-date. The smart contract provides an unchangeable and impenetrable record of trust-related data. The BRGTL smart contract makes use of the distributed ledger to guarantee the distribution of trust information among nodes [82]. It minimizes the likelihood of an SPOF and increases the trust-management system’s overall resilience. The BRGTL serves as a historical reference for evaluations of trust. It keeps an updated record of trust values, which enables the system to monitor changes over time and spot patterns in device behavior.
3. Trust Updating at Fog Layer
  In the proposed framework, trust updating is carried out by the fog layer. Real-time updates to the trust values of nodes at the device layer are continually revising the trustworthiness of devices within the smart healthcare CPS. Every time a new device connects to the network or after a predetermined amount of time, the trust values are routinely updated by the GBG-RPL [89]. However, the device or subsequent trust calculation is assigned a minimum score of acceptable trust upon its initial request to join the network. The fog layer communicates with the device layer to share the updated trust values [61]. This communication ensures that devices at the device layer are informed of changes in trust assessments and can initiate action accordingly.
4. Trust Management to Prevent Blackhole and Greyhole Attacks
  The GBG-RPL ensures that the system can adapt to changes in the network’s behavior. If a node starts behaving maliciously, the GBG-RPL will reflect this anomaly, triggering alerts and enabling the system to take preventive actions. The BRGTL smart contract on the blockchain stores a secure and tamper-resistant record of trust values. Even if a greyhole or blackhole attack attempts to manipulate local trust values, the immutable nature of the blockchain prevents the compromise of the overall trust assessment [82]. The BRGTL smart contract acts as a decentralized source of trust. Nodes in the device layer can verify trust values against the historical records stored in the blockchain, ensuring that trust information is consistent and not manipulated by attackers.

## 6. System Architecture

**Figure 4:**
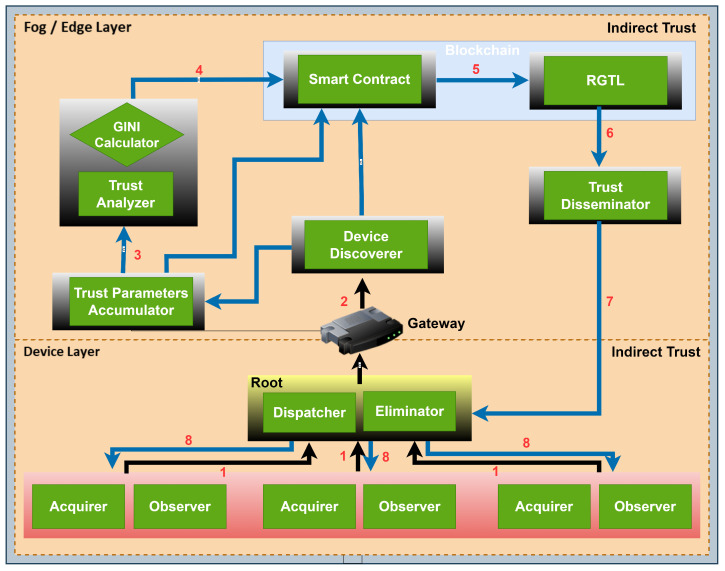
System architecture.

The proposed system architecture is depicted in Figure 4, which provides a detailed representation of all the components. The proposed methodology has been named the Gini index and blockchain-based framework for detecting blackhole and greyhole attacks and is referred to as GBG-RPL in this study. To execute the simulations for this study, three types of nodes were considered—the fog node, sink node, and child node. The Cooja simulator was used under predetermined parameters. The child nodes at the device layer keep an eye on their neighbors’ packet-forwarding behavior along with the energy use, message overhead, and end-to-end delay. These parameters are sent to the fog server at predetermined intervals via the sink/root node. The fog node calculates the trust value using the Gini index, which is subsequently saved in the BRGTL. The global trust list is kept up-to-date as a decentralized ledger that is distributed to child nodes for attack detection and decision making as to whether to accept a node’s request to join them as a child. As a mitigating mechanism, a node would likely be isolated and blacklisted from the network if the Gini index value were >0.5. The proposed system architecture is divided into different modules, as described below:

1. Observer and Acquirer/Direct Trust Collector: The sink/root node and the resource-constrained nodes are the two types of nodes present in the device layer. The computing, storage, and energy capacities of these nodes are constrained. According to the RPL architecture, these nodes are either parent or child nodes. The parent nodes carry out the functions of the observer and acquire trust parameters. In the proposed architecture, these nodes are performing two functions named observer and acquirer. The parent nodes observe the parameters of their neighbor nodes and child nodes, such as the packet loss, energy usage, end-to-end delay, and message overhead during normal RPL operations of the CPS, and pass these parameters to the sink/root node. The acquirer function collects the parameters, while the observer function monitors the behavior of the nodes. In the event that a child node is flagged as malicious in the global trust list, the parent nodes remove that child node from the CPS network. The RPL becomes unstable, and its rebuilding process is initiated whenever a node enters or exits the network. Following that, the CPS network resumes its regular RPL activities.
2. Dispatcher–Eliminator: As per the proposed framework, the sink/root node has more processing power, storage, and enhanced energy backup in comparison to other nodes at the device layer. The root node has two primary functions, including dispatcher and eliminator. To connect the device layer and the fog layer, the sink/root node acts as a link. As all traffic between the fog layer and device layer is routed through the sink node, the root node also serves as the cluster head. The dispatcher function gathers all trust parameters received from the parent nodes at the device layer of the CPS network and forwards the same to the fog server at the fog layer. Furthermore, the eliminator function receives the revised BRGTL from the fog layer and forwards or broadcasts downwards to all member nodes at the device layer. The root itself is not making any decisions, and it is only receiving and forwarding the traffic between the device and the fog layer.
3. Device Discoverer: The device discoverer module is a vital component of the proposed framework, operating at the fog layer to keep track of all nodes entering and leaving the CPS network. Its main function is to maintain a comprehensive record of node activity, which is essential for network management and security. This module forwards its processed device list to the trust parameter accumulator module and blockchain ledger updater (smart contract) module. A comprehensive working overview of the device discoverer module is described below.
  (a) Registering devices: The device discoverer is responsible for registering a newly discovered node that wants to become part of the CPS network. It gathers pertinent data about the new node, such as its exact location, identification information (such as MAC address and node ID), and additional characteristics.
  (b) Authenticating devices: The device discoverer verifies the legitimacy of each node’s request through authentication procedures before having registered it. This is an essential step in preventing malicious or unauthorized devices from pairing with the network.
  (c) Updating BRGTL: Following a successful device registration and authentication, the device discoverer modifies the global trust list that the blockchain-based trust-management system keeps up-to-date. To monitor its actions and contributions to the network, the details of the new node are added to the list, along with any available trust metrics.
  (d) Tracking node activity: The device discoverer continuously monitors the CPS network for any node activity, including nodes entering and leaving the network.
  (e) Monitoring node status: The device discoverer regularly checks the status of registered nodes to ensure their proper functioning and responsiveness.
  (f) Handling departure of nodes: The device discoverer deletes a node from the list of active nodes and modifies its status in the BRGTL when it departs the CPS network.
  (g) Detecting malicious nodes: An important function of the device discoverer is to identify potentially malicious nodes in the network by constantly tracking node behavior and activity. The trust analyzer receives reports of any odd or unusual behavior for additional analysis and suitable action.
  (h) Logging: Every node activity, such as fresh node registrations, exits, and any unusual behaviors found, is recorded by the device discoverer.
  (i) Integration with other modules: The device discoverer exchanges data regarding node actions and trust status with the other elements of the framework, including the trust analyzer and calculator and the eliminator. It offers vital information for processes related to threat prevention and trust-based decision making.
  By functioning as a comprehensive node activity tracker and coordinator, the device discoverer module enhances the overall security and management of the CPS network. Its ability to identify and respond to new node entries and departures contributes to the dynamic and adaptive nature of the framework in detecting and mitigating blackhole and greyhole attacks.
4. Trust Parameter Accumulator: The trust parameter accumulator operates in the fog layer to collect and aggregate trust parameters received from the device layer. Its primary function is to gather all relevant trust-related data from the device layer (root node) and combine them for further processing by the trust analyzer and calculator module. The processed trust parameters are also forwarded to the blockchain ledger updater module. The trust parameter accumulator module acts as a vital intermediary between the device layer and the trust analysis components at the fog layer. By efficiently collecting, aggregating, and pre-processing the trust parameters, it enables accurate and timely evaluation of node behavior.
5. Trust Analyzer and Calculator: At the fog layer, fog servers with strong computational capability are set up. On the basis of the received parameters, the trust calculator executes Gini index-based logic and calculates the Gini value, which determines whether the node’s behavior is malicious or normal. If the Gini value is >0.5, then the behavior is attributed to an attack, and if the Gini value is <0.5, then the node is treated as a normal node. However, it is noteworthy that for critical/sensitive requirements, only nodes with lower Gini values, preferably closer to zero, would be allowed to form the DODAG.
  It is possible to tell the difference between nodes with a regular distribution pattern and those with malicious intent by looking at their distribution patterns. The value 1 can be achieved through either a uniform or an uneven distribution. As shown in Table 6, a node-rating threshold is determined using Gini logic. The main aim of these thresholds is to isolate harmful nodes from the rest of the network. Only nodes that have passed this test are allowed to take part in routing decisions. To provide more elaborate details, the different steps and processes involved in this module are described below.
  (a) Trust parameter collection: The trust analyzer and calculator module receives trust parameters from the trust parameter accumulator module and parent nodes at the fog layer.
  (b) Trust parameter normalization: Before calculating the Gini index, the trust parameters are normalized to bring them to a consistent scale.
  (c) Trust score calculation: A trust score is a numerical indicator of a node’s reliability or trustworthiness in a network. Trust scores are essential for evaluating node behavior in the context of a CPS, especially when it comes to identifying and reducing the possibility of malicious nodes. The suggested method uses trust scores to assess nodes’ credibility according to trust parameters. In a CPS network, the overall “trustworthiness inequality” between nodes can be evaluated using the Gini index. It offers a tool for locating potentially dangerous nodes or ones that drastically depart from the norm when paired with trust scores. Algorithm 1 explains how the suggested method calculates the trust score.
  (d) Gini index calculation: The Gini value for every node under observation is determined by the trust analyzer and calculator using logic based on Gini indexes. To calculate the Gini index, add up all of the trust parameter pairs’ absolute differences, then divide the total by the number of pairs. The suggested method of Gini index computation is described in Algorithm 1.
  (e) Threshold setting for malicious behavior: After calculating the Gini value for each node, the trust analyzer and calculator sets a threshold value to distinguish between normal and malicious behavior. The threshold value is set at 0.5, but this can be adjusted based on the specific requirements and characteristics of the CPS network.
  (f) Node classification: Nodes with Gini values above the threshold (>0.5) are classified as exhibiting potentially malicious behavior, indicating a higher degree of parameter inequality. Nodes with Gini values below the threshold (<0.5) are considered normal nodes, exhibiting a more uniform distribution of trust parameters.
  (g) Formation of DODAG based on trust: To ensure the security and efficiency of the CPS network, the trust analyzer and calculator may influence the formation of the destination-oriented directed acyclic graph (DODAG) based on trust values. Nodes with lower Gini values, preferably closer to zero, are given priority in forming the DODAG, especially for critical/sensitive tasks or routing decisions.
  (h) Trust updates and periodic review: The trust analyzer and calculator periodically reviews the trust values based on the updated trust parameters received from the device discoverer and parent nodes.
6. Blockchain Ledger Updater for BRGTL: The blockchain ledger updater module, functioning as a smart contract in the fog layer, plays a critical role in the proposed methodology by leveraging blockchain technology to update and maintain the BRGTL. This module integrates inputs from various components, including the device discoverer module, trust parameter accumulator module, and trust analyzer and calculator module, to ensure the trustworthiness and reliability of the CPS network. The fog server implements blockchain technology to update and keep track of the BRGTL. When a new node requests to join the network, a new entry is added here. Through the use of the sink node, the parameters of the parent node are transmitted to the fog server. A new entry is added to the blockchain if the node’s information is not already included there; otherwise, the list is not updated. Similarly, the list is updated during regular operations if there are any deviations in metrics like packet loss, end-to-end delay, message overhead, or energy consumption. Below is an elaborate description of the functionality of the blockchain ledger updater module as a smart contract.
  (a) Smart contract deployment: The fog server deploys the smart contract in the selected blockchain network. The smart contract’s code contains the logic for managing the global trust list and processing trust-related data. The deployment process involves uploading the compiled smart contract code to the blockchain platform through relevant APIs or tools.
  (b) Receiving trust parameters: The blockchain ledger updater module receives trust parameters from multiple sources, including the device discoverer module, which keeps a record of all nodes entering and leaving the CPS network. Trust parameters from various nodes, such as packet loss, end-to-end delay, message overhead, and energy consumption, are gathered and sent to the blockchain ledger updater for further processing.
  (c) Global trust list update: Based on the trust evaluation results, the blockchain ledger updater module updates the global trust list (BRGTL) in the blockchain. The list contains entries for each node, reflecting their trust values and other relevant information. New nodes requesting to join the network have their entries added to the BRGTL.
  (d) Immutability and consensus: The smart contract ensures that the global trust list on the blockchain is immutable and tamper-resistant. Once trust data are recorded, they cannot be altered or deleted, ensuring the integrity and reliability of the trust registry.
  (e) Decentralization and transparency: As a smart contract in the fog layer, the blockchain ledger updater operates in a decentralized manner, thus removing the need for a central authority figure.
  (f) Logging and event handling: The module logs and handles events related to trust parameter updates and BRGTL modifications.
  By efficiently updating the BRGTL in the blockchain, this module ensures a secure and decentralized trust-management system for the CPS network.
7. Trust Disseminator: The trust disseminator is responsible for distributing the BRGTL from the fog layer to the device layer of the CPS network. The revised list is sent to the disseminator module after the trust has been determined and the blockchain has been updated. The disseminator module transmits the updated list to the device layer through the sink node, which is the third function of the fog server. This module is just in charge of trust distribution to the device layer and does not perform any calculations. A thorough explanation of the trust disseminator module’s working is outlined below:
  (a) Receiving BRGTL: After trust calculations are completed and the blockchain is updated with the latest trust values for each node, the blockchain ledger updater forwards the BRGTL to the trust disseminator module.
  (b) Sending BRGTL to device layer: When the data have been prepared, the sink node allows the trust disseminator to send the BRGTL to the device layer. Because it acts as an intermediary between the fog and device layers, the sink node is the most appropriate option for sharing trust data.
  (c) BRGTL distribution: The primary function of the trust disseminator is to use the root node to transmit the trust data to each node in the device layer. It guarantees that each node in the network is aware of the reliability of its nearby peers and other nodes.
  (d) Synchronizing trust update: To guarantee that each node has access to the most recent trust values, the trust disseminator module regulates the trust changes in all nodes in the device layer through the root node.
  (e) Trust-based decision making: Nodes can decide how to interact with other nodes based on trust when trust information is quickly disseminated. During the RPL process, nodes are able to employ this information to assess the reliability of prospective parents or children.
  The trust disseminator module serves as a critical link between the fog layer, where trust evaluations occur and the BRGTL is updated, and the device layer, where trust information is required for network operations and decision making. By efficiently distributing the updated trust values, this module enhances the trust-management system’s effectiveness. It contributes to the successful detection and mitigation of blackhole and greyhole attacks in the GBG-RPL framework.
8. Eliminator: The eliminator module is a crucial component in the GBG-RPL methodology for taking action based on the BRGTL received from the trust disseminator module. This module ensures the containment of malicious nodes and maintains the integrity of the CPS network. Upon receipt of the BRGTL from the disseminator, the sink node forwards the same to the parent nodes. The parent node initiates action to isolate or deny access to the network to the malicious node. After attacker containment, the RPL is rebuilt, and subsequently, routine CPS network operations are started. Similarly, if a new node is joining the network, the RPL is rebuilt, followed by routine CPS network operations. The eliminator module’s functionality is critical in maintaining a secure and reliable CPS network. By promptly isolating malicious nodes and incorporating new nodes through RPL rebuilding, this module ensures that the network remains resilient to blackhole and greyhole attacks.

## 7. CPS Architecture in GBG-RPL

**Figure 5:**
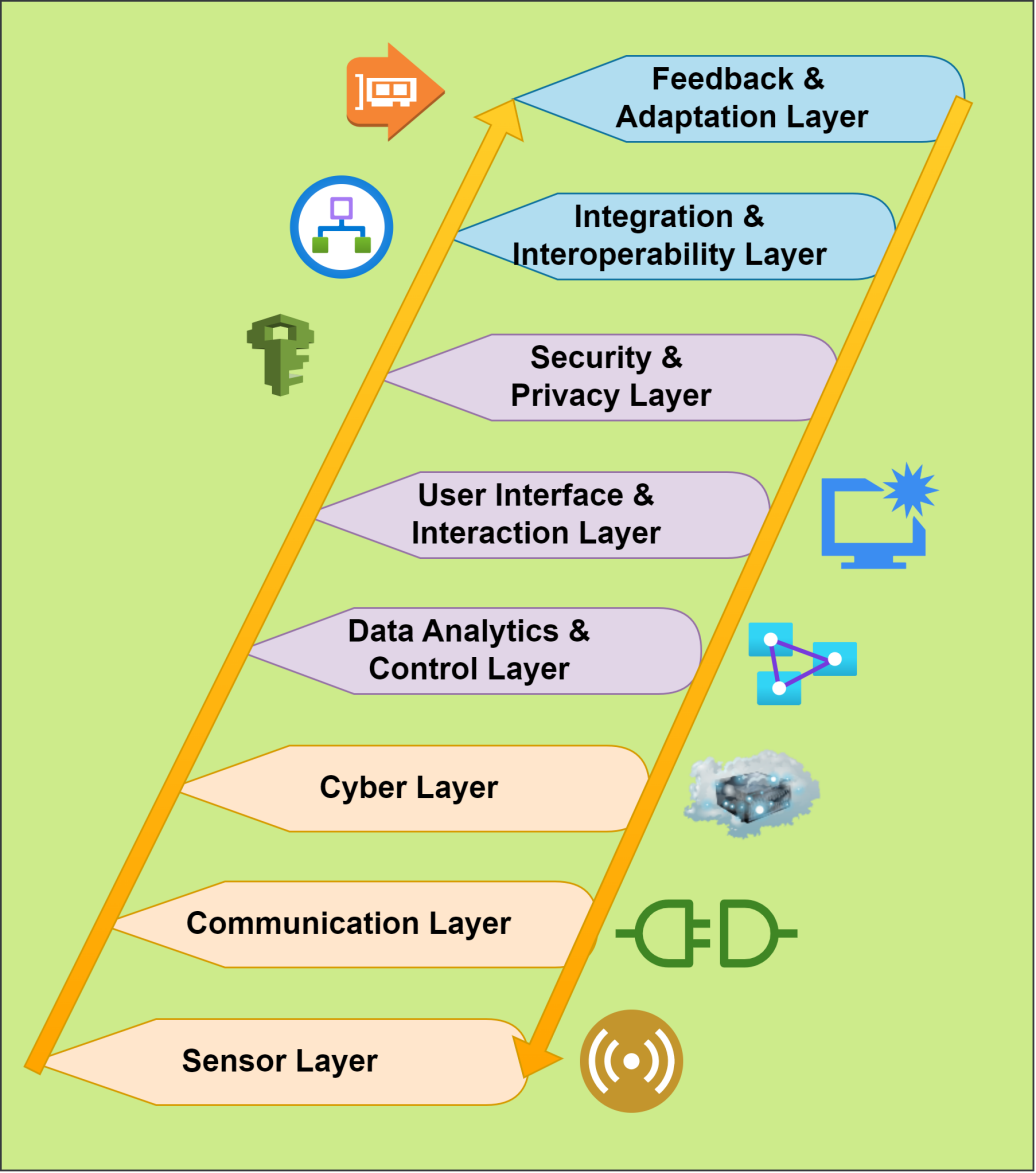
A layered CPS architecture.

The CPS architecture in the proposed methodology comprises several interconnected layers that facilitate communication and coordination between physical and cyber components, as shown in Figure 5. A high-level representation of the architecture is given below:

1. Sensing Layer: The sensing layer serves as the core of the CPS architecture. It is made up of a number of sensors and actuators that are placed throughout the physical environment (e.g., smartwatches and fitness bands) to gather information from the patients [90]. The sensors record critical data from the patients related to location/motion, blood pressure, heartbeat, and sugar levels for central monitoring. The actuators interact with the physical world by carrying out operations according to commands from the cyber system (e.g., implantable devices like pacemakers and insulin pumps function as adjusted by the practitioners).
2. Communication Layer: Data transmission between the sensing layer (devices on the patients) and the cyber layer (servers and central control/monitoring at hospitals) is carried out by the communication layer [91]. It consists of networks, gateways, and communication protocols that make it easier for data to be sent from sensors and actuators to cyber components for analysis and decision making.
3. Cyber Layer: The cyber layer applies control algorithms, data analytics, and decision-making procedures to the data it receives from the sensing layer. This layer consists of computer hardware such as edge servers, cloud servers, and control systems that process the incoming data to produce useful insights and coordinate the operations of the physical layer [92].
4. Data Analytics and Control Layer: Data analysis and thoughtful decision making are the main functions of this layer [93]. This layer processes the raw data gathered from the sensing layer to discover useful patterns and make informed decisions.
5. User Interface and Interaction Layer: Users can interact with the CPS system through the user interface layer [94]. Applications, dashboards, and visualizations are included that let users control parameters, monitor system performance, and input data to adjust the function of the CPS device.
6. Security and Privacy Layer: The safety and privacy of the CPS must be guaranteed at all costs. This layer protects the CPS against online threats and unauthorized access through a variety of security methods, encryption protocols, access-control systems, and authentication procedures [24].
7. Integration and Interoperability Layer: CPSs frequently require the integration of several parts and systems from diverse vendors. Smooth coordination inside the CPS system is made possible by the integration and interoperability layer, which enables seamless communication and cooperation between these many components [95]. In the proposed methodology, the device ID along with the trust parameters are stored in the device acquirer and blockchain-based revised global trust list (BRGTL).
8. Feedback and Adaptation Layer: This layer contains feedback loops and adaptive control systems that let the CPS devices react quickly to commands given by the central control/monitoring systems [96].

### Workflow of Proposed Model (GBG-RPL)

**Figure 6:**
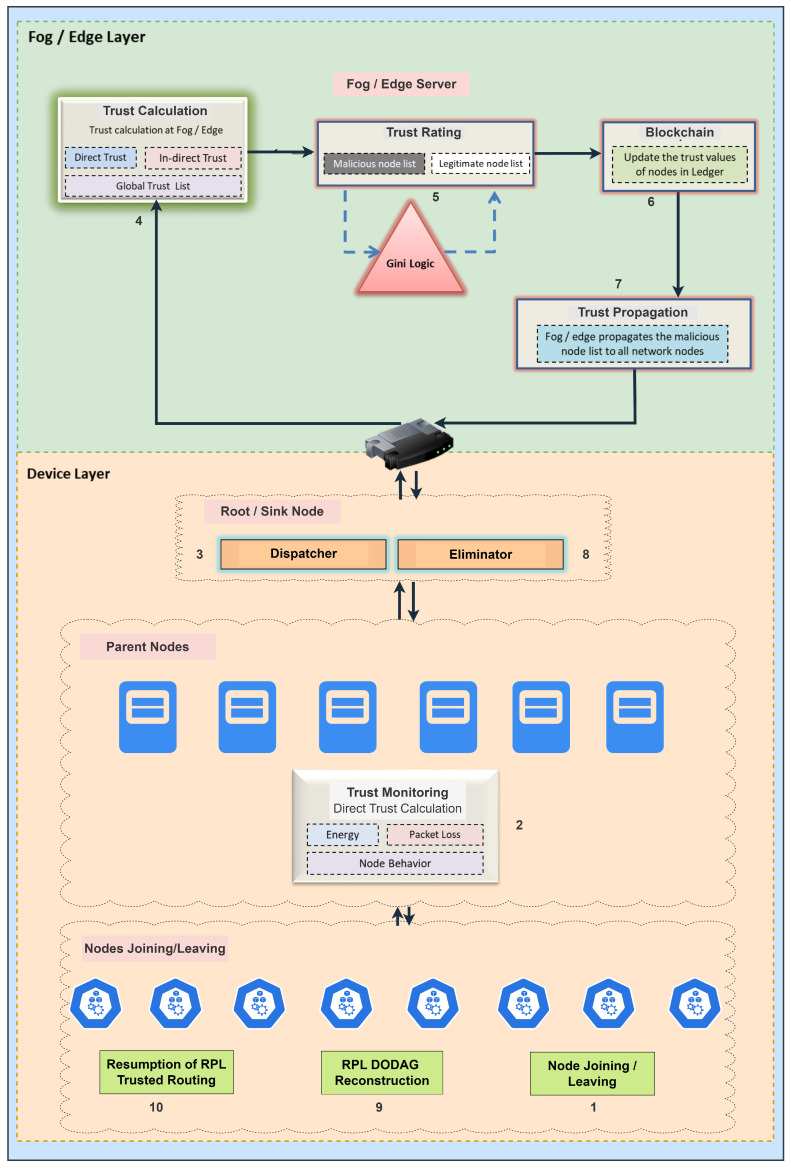
The proposed methodology.

The step-by-step workflow of the proposed methodology is depicted in Figure 6. The figure depicts a fog server deployed at the fog layer, a root node at the device layer, and parent/child nodes at the device layer. A detailed description of each step is given below:

1. When a member node departs the CPS network or a new node enters the network, the RPL DODAG reconstruction begins.
2. Parent nodes keep an eye on the trust parameters of their child nodes and neighbors. Dropped packets, energy consumption, latency from start to finish, and message size are all the parameters to keep an eye on. It is performed at the device layer and is sometimes referred to as “direct trust calculation”. Except for the sink node, all other nodes at the device layer have limited resources.
3. Each child node reports its trust parameters to its parent, which then shares them with the sink. Except for transmitting the parameters to the fog server, the sink node does nothing else. This means that the sink node is connecting the device layer and the fog layer. The update of trust parameters is sent from the device layer to the fog layer every 10 milliseconds (ms). At the same time, the trickle timer for considering a packet to be dropped/lost is set at 5 ms.
4. The fog server is deployed at the fog layer. It has high computational and storage capabilities and, therefore, is assigned many tasks or functionalities for execution. The first function is to maintain a global trust list, which is updated on the basis of direct and indirect trust calculations. Information regarding all member nodes resides in the global trust list.
5. The second functionality is to calculate the trust value for a node by applying the Gini logic. The resulting value of the Gini logic falls between 0 and 1, where 0 denotes a fully trusted node while 1 denotes a fully compromised node. Thus, the behavior of a node is declared either malicious or normal as per the trust value calculated.
6. Once the trust value has been calculated, the blockchain smart contract is implemented to update the ledger/database, which maintains the global trust list. The ledger is updated only when there is the addition of a new node or the trust status of the node changes.
7. After the update of the database, the BRGTL is disseminated from the fog layer to the device layer.
8. The sink node receives the updated BRGTL, which is then sent to the parent nodes so that the malicious node can be removed or a new node can be allowed to join the network.
9. The RPL DODAG is rebuilt in the event that a malicious node is removed from the CPS network, a new node joins the network, or a member node leaves the network.
10. After the reconstruction of the RPL DODAG, routine RPL network operations are resumed.

## 8. Methods for Designing, Testing, and Deploying the GBG-RPL Framework in Smart Healthcare CPS

The methods for designing, testing, and deploying the GBG-RPL framework can be categorized into several types, as described below:

1. Designing the GBG-RPL Framework
  The design of the GBG-RPL framework incorporates various methods to ensure a robust and adaptable system. Mathematical design plays a key role, involving the formulation of the Gini index-based algorithm along with trust metrics [97]. By leveraging mathematical principles, this method defines the trust assessment and adaptation mechanisms within the framework. Additionally, interdisciplinary collaboration is a cornerstone of the design process, fostering cooperation between cybersecurity and healthcare experts [98]. This collaborative approach ensures a holistic design that aligns with both mathematical rigor and the real-world requirements of healthcare systems. Furthermore, allowing tailoring of the GBG-RPL to specific characteristics of smart healthcare systems enables the customization of the parameters and algorithm based on the unique deployment requirements.
  To mitigate the security risks associated with the implementation of the proposed framework in a smart healthcare system, a comprehensive set of measures is undertaken. First and foremost, a decentralized architecture is adopted to distribute trust-management functions across multiple nodes, reducing the vulnerability to centralized attacks [85]. In the proposed framework, the trust calculations, the ledger in the form of BRGTL, device registration, and smart contracts have been implemented in the fog servers, which are deployed in the fog layer. The implementation of the fog layer mitigates the direct exposure of the trust calculation and database from the device layer where the resource-constrained devices are exposed to the attackers [17]. Regular updates and security audits would be conducted for the blockchain network and smart contracts to address vulnerabilities and ensure the robustness of the system. Scalability challenges are managed using a layered architecture, where multiple fog servers are implemented to accommodate large numbers of nodes, large data volumes, and geographic expansion. To navigate regulatory complexities, close collaboration with legal experts would ensure that the proposed framework aligns with relevant healthcare data protection regulations [99]. Thorough testing and validation during integration, the involvement of cybersecurity experts, and seamless communication between the proposed framework and existing components would address concerns related to integration complexity. These multifaceted measures collectively contribute to a resilient security infrastructure for the smart healthcare system.
2. Testing the GBG-RPL Framework
  The evaluation of the GBG-RPL framework involves two distinct testing methodologies to ensure its effectiveness and reliability. Simulation testing is conducted using the Cooja simulator, wherein trust parameters and attack configurations are systematically varied. This process helps identify potential issues and assesses the framework’s scalability and adaptability under different conditions [100]. The second part complements the simulation testing with real-world testing, which takes place in actual smart healthcare environments. The evaluation process offers an in-depth understanding of the framework’s effectiveness across diverse scenarios by utilizing the simulations and real-world testing, which add to the framework’s robustness and applicability in real-world healthcare settings.
  (a) Deployment Methods for the GBG-RPL Framework
    The implementation of the GBG-RPL framework necessitates the smooth integration of GBG-RPL into the existing infrastructure of intelligent healthcare systems [9]. This integration guarantees compatibility and cooperation with other components. Another crucial component of the deployment strategy is user awareness and training, which involves teaching administrators and users about GBG-RPL’s features and security precautions. These methods enable modifications in response to real-time input and evolving needs, guaranteeing the framework’s flexibility and long-term efficacy in smart healthcare scenarios.
  (b) Additional Considerations
    The implementation of the GBG-RPL framework employs a thorough and repetitive methodology to guarantee ongoing improvement and peak efficiency. Using an iterative development cycle facilitates continuous improvement by incorporating knowledge gained from the deployment and testing phases into later iterations [101]. In order to guarantee alignment with user needs, interactive development methods involve stakeholders such as administrators, cybersecurity specialists, and healthcare professionals. Security audit and compliance measures are implemented to conduct thorough security audits, ensuring adherence to industry standards and regulations [102]. User feedback is actively sought to inform design improvements, adopting a user-centric design approach that ensures the uninterrupted availability of healthcare services. Additionally, the deployment emphasizes the adoption of best practices in cybersecurity and healthcare data management, drawing insights from successful implementations in related domains [103].

## 9. Experimentation

The purpose of the experiments is to assess the performance and efficacy of the GBG-RPL approach, which includes the Gini index and blockchain technology, in identifying and mitigating blackhole and greyhole attacks in a CPS network. Utilizing a Linux-based platform, the Cooja network simulator was used to implement the trust mechanism. The Cooja simulator offers an appropriate setting for simulating and assessing CPS networks. Table 7 includes all simulation parameters.

### 9.1. Hardware Requirements

To ensure optimal performance and efficient execution of the experimental setup, the following hardware specifications were used during experiments. A PC with a multi-core processor with a clock speed of 2.4 GHz, 8 GB RAM, and 128 GB SSD with an additional 512 GB HDD was used to handle the computational workload effectively. The experimental setup is designed to run on a Linux-based platform; therefore, a Linux distribution such as Ubuntu, Fedora, or Debian is ideal for compatibility and optimal performance. These hardware parameters ensured the efficient execution of the simulations. In this experiment, the Cooja simulator, running on a Linux-based platform, provided the necessary tools and resources for simulating and evaluating the GBG-RPL methodology in a CPS network. Furthermore, three types of nodes used during the simulation are described in Table 8.

### 9.2. Libraries and Frameworks

The Contiki operating system (OS) and Contiki programming language, which were created especially for CPS devices with limited resources, were used in the experimental setup. The features and protocols required to simulate CPS devices inside the Cooja environment were given by Contiki. The Cooja simulator used a variety of frameworks and libraries, including:

1. RPL Classic: The uIP stack has capabilities including packet processing, routing, and communication protocols and supports IPv6. It ensured that the CPS network was routed effectively and consistently, which assisted in the evaluation and realistic behavior of the trust mechanisms.
2. Managing Trust: A trust-managing library (TinyDTLS) was employed in the GBG-RPL technique to facilitate the detection of blackhole and greyhole attacks. With the help of these libraries, trust values could be managed and calculated while accounting for a number of variables, such as message overhead, energy consumption, packet loss, and end-to-end delay.
3. Blockchain Library (web3.js): The web3.js package was used to include blockchain technology.

### 9.3. Simulation Environment

In the course of the research, a CPS network comprising thirty nodes in the device layer, one sink/root node, and one edge server in the fog layer was used. This network architecture, which reflected a typical configuration for CPS networks, made it possible to evaluate the GBG-RPL technique in a practical setting. Various communication factors were taken into consideration to assess the blackhole and greyhole attack detection and mitigation. For the purpose of calculating and analyzing trust, these parameters, including energy consumption, packet drop, end-to-end delay, and message overhead, were gathered from the device layer and sent through the root node to the fog layer. The GBG-RPL methodology used the web3.js framework to combine blockchain technology and the Gini index calculation in the fog layer.

In contrast, the Gini index functions as a gauge of trustworthiness based on communication metrics. Energy consumption, packet drop rate, end-to-end delay, message overhead, and attack-detection rate were among the performance parameters that were measured and studied during the trials. These measurements revealed how well the GBG-RPL methodology worked to identify and stop blackhole and greyhole assaults.

The elaborate experimental setup incorporated the use of the Cooja simulator to apply and assess the GBG-RPL approach within a CPS network and the results were compared to a reference technique named BCPS-RPL. A number of simulation settings were taken into account throughout the setup, including the attacker-to-malicious-node ratio, receive-to-transmit ratios, interference-to-transmission distances, and use of the RPL routing protocol. The 60-minute simulation time frame allowed for a full evaluation of the trust mechanisms’ effectiveness over a lengthy period. The effectiveness of the GBG-RPL methodology in identifying and mitigating blackhole and greyhole attacks was assessed by collecting and examining performance indicators. The incorporation of the Gini index and blockchain technology improved the system’s reliability and security. In order to increase the overall dependability and trustworthiness of the healthcare CPS, the experimental setting aimed to contribute to the development of secure and reliable trust-based procedures.

## 10. Results and Discussion

The evaluation measures are discussed in this section, along with the related graphs. The descriptions of the graphs are included in each of the subsections.

### 10.1. Packet Loss Ratio

**Figure 7:**
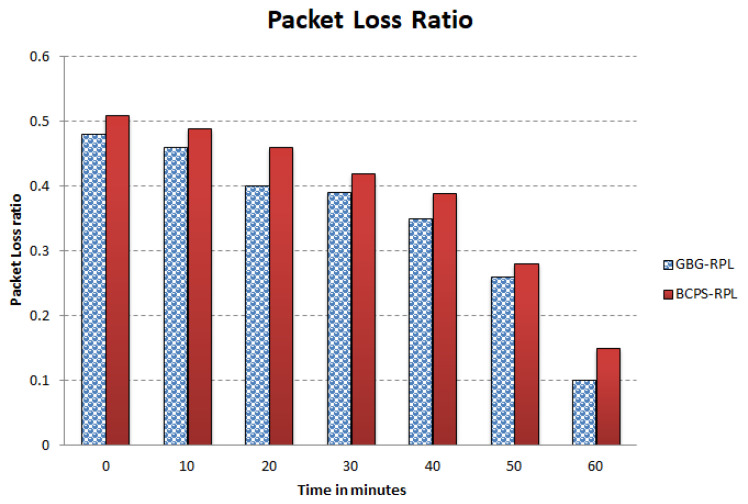
Packet loss ratio.

The packet loss ratio (PLR) is a network performance metric that measures the proportion of packets that are lost or discarded during data transmission across a network. The packet loss ratio over time for the GBG-RPL and BCPS-RPL mechanisms is shown in Figure 7.

For both the GBG-RPL and BCPS-RPL processes, Figure 7 shows a declining trajectory of the packet loss ratio with time. The GBG-RPL mechanism consistently maintains a marginally lower packet loss ratio than the BCPS-RPL mechanism at each time interval, demonstrating a higher individual packet transmission efficiency. The packet loss ratio of the GBG-RPL is better than that of BCPS-RPL due to its better detection capability to identify and isolate the blackhole and greyhole nodes in the CPS network. Initially, there is less difference in the PLR of both techniques; however, the GBG-RPL exhibits further improvement in PLR. The high PLR of BCPS-RPL is due to two major factors. Firstly, it is due to the large volume of packet transmission in the device layer, which results in network congestion. Secondly, it is due to delayed detection of the malicious nodes, which causes packet drop. In the case of the GBG-RPL, the improvement is attributed firstly to the shifting of all trust calculations from the device layer (sensor nodes) to the fog layer (fog server), secondly to the careful selection of the significant trust parameters, and thirdly to the trickle timer.

### 10.2. Energy Consumption

**Figure 8:**
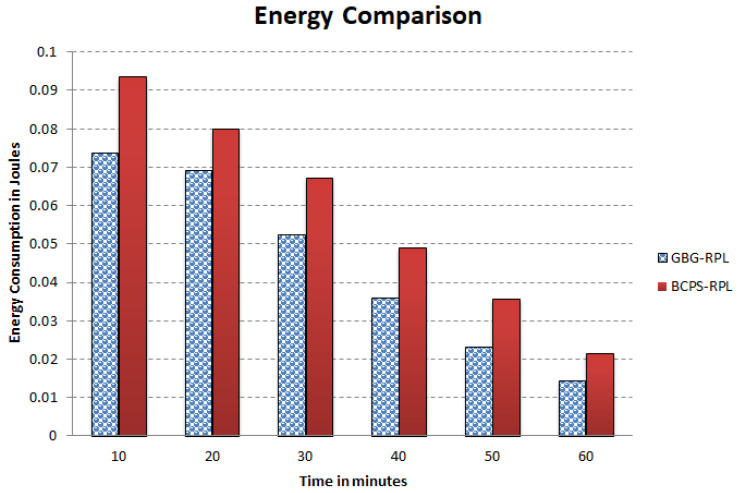
Energy consumption.

Energy consumption refers to the amount of energy used or consumed by a device over a specific time. Figure 8 shows the energy consumption of the GBG-RPL mechanism and the BCPS-RPL mechanism over time.

Figure 8 illustrates a trend for both the GBG-RPL and BCPS-RPL mechanisms to consume less energy with time. The GBG-RPL displays less energy consumption over the simulation time, which results in enhanced network life. This means more availability of the CPS network. In both techniques, initially, the nodes consume more energy as the system is initialized and trust in the system is established. However, in GBG-RPL, the nodes are only observing and forwarding the trust parameters to the root, whereas, in BCPS-RPL, the nodes are observing the neighbor nodes for their behavior and also calculating the trust score, which increases energy consumption. This trend of high energy consumption in BCPS-RPL and low energy consumption in GBG-RPL is evident throughout the simulation time. Furthermore, once the attack is injected into the CPS network, the GBG-RPL takes less time to identify the attacker as compared to the BCPS-RPL, which further contributes to the energy savings of the sensor nodes.

### 10.3. Average Residual Energy

**Figure 9:**
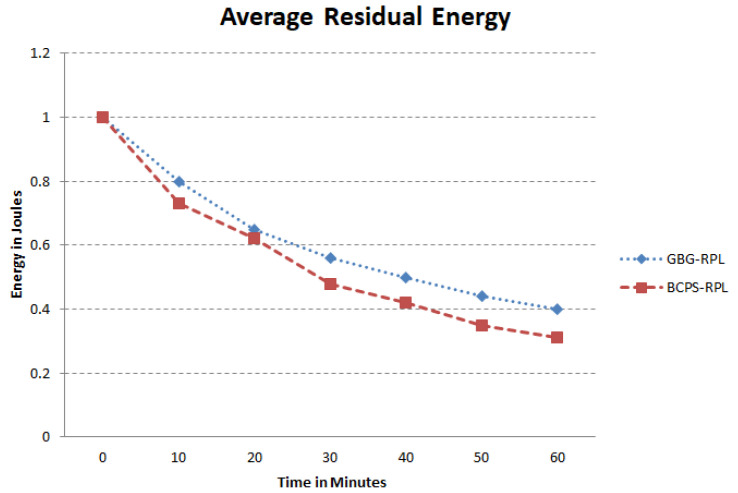
Average residual energy.

Average residual energy is the average amount of energy remaining in a group of wireless nodes or devices after a certain time or a series of operations. The average residual energy with respect to time for the GBG-RPL and BCPS-RPL mechanisms is shown in Figure 9.

For both the GBG-RPL and BCPS-RPL processes, Figure 9 shows the average residual energy’s declining trend over time. It illustrates that as time goes on, both mechanisms’ energy levels drop, showing that the CPS is using up its energy reserves. The GBG-RPL displays a high average residual energy throughout the simulation time, resulting in an enhanced network life. This means more availability of the CPS network. In both approaches, the nodes initially consume more energy during system initialization as trust in the system is established. However, in GBG-RPL, nodes solely observe and forward trust parameters to the root. In contrast, BCPS-RPL nodes not only observe neighbor behavior but also calculate trust scores, leading to increased energy consumption. This pattern of low average residual energy in BCPS-RPL and high average residual energy in GBG-RPL persists throughout the simulation. Additionally, when attacks are introduced into the CPS network, GBG-RPL detects the attacker faster than BCPS-RPL, contributing to energy savings for sensor nodes and improving network longevity.

### 10.4. End-to-End Delay

**Figure 10:**
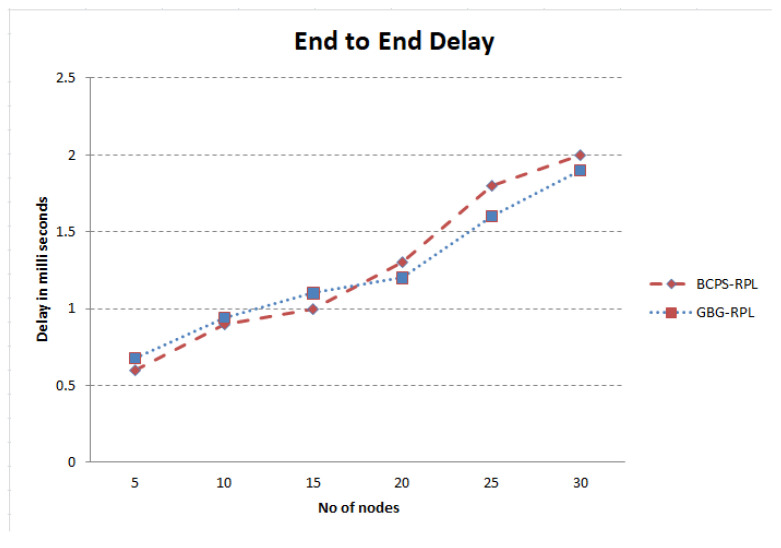
End-to-end delay.

The time it takes for data packets to move from source to destination within the network is revealed by analyzing the end-to-end delay results for the GBG-RPL and BCPS-RPL methodologies. The relationship between the number of nodes and the end-to-end delay for both the BCPS-RPL and GBG-RPL methods is depicted in Figure 10.

The relation between the number of nodes and the end-to-end latency for both the BCPS-RPL and GBG-RPL mechanisms in the CPS is shown in Figure 10. It proves that for both systems, the end-to-end delay grows with the number of nodes. The outcomes show that the GBG-RPL mechanism outperformed the BCPS-RPL mechanism in terms of end-to-end delay. Initially, the BCPS-RPL has a smaller end-to-end delay, which is due to the fact that the initial trust is being calculated in the device layer, and this trend continues until 15 min into the simulation. Similarly, the GBG-RPL displays a high end-to-end delay at the initialization until 15 min of simulation as the packets are traveling from the device layer to the fog layer. However, once the trust has been established and attacks are induced, the trends for both techniques change and the GBG-RPL starts performing better. The main reason is that in BCPS-RPL, the nodes are continuously performing trust calculations at the device layer, and repetitive trust calculations have induced congestion at the nodes in the form of longer packet queuing, processing delays, propagation delays, and transmission delays. The end-to-end delay is further increased once the attacks are induced, and packets are retransmitted frequently. In contrast, in the case of the GBG-RPL, once the trust has been established in the network, only trust parameters are being forwarded from the device layer to the fog layer, which reduces the network congestion at the device layer. Furthermore, once the attacks are induced in the network, the attackers are detected in a timely manner, and only information related to the malicious node is propagated from the fog layer to the device layer. Therefore, the GBG-RPL, in the longer run, reduces its end-to-end delay, which improves the network efficiency.

### 10.5. Attack-Detection Rate

**Figure 11:**
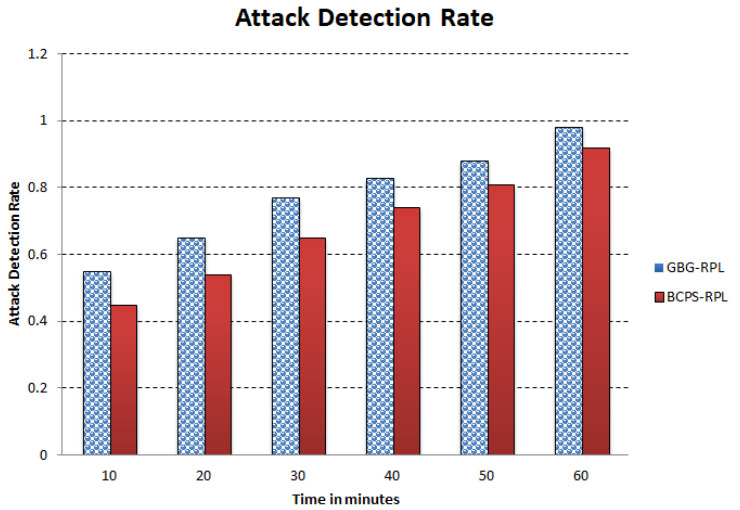
Attack-detection rate.

The attack-detection rate measures the proportion of actual attacks or malicious events that are correctly detected and flagged by the security mechanism as positive. The attack-detection rates for the BCPS-RPL and GBG-RPL mechanisms within a CPS are shown in Figure 11.

The bar graph shows an increasing trend in attack-detection rates over time for both the BCPS-RPL and GBG-RPL techniques. The findings show that, in terms of attack=detection rate, the GBG-RPL mechanism beat the BCPS-RPL mechanism. The attack-detection rate is directly dependent upon the identification of the malicious nodes. Once the attacker is removed from the CPS network, the procedure is finished. Throughout the simulation period, the GBG-RPL detects attacks more frequently than the BCPS-RPL. This is because the trickle timer’s configuration and trust parameters were chosen with care. As a result, the GBG-RPL has a higher overall attack-detection rate, which improves network life and reliability.

### 10.6. Attack-Detection Time

**Figure 12:**
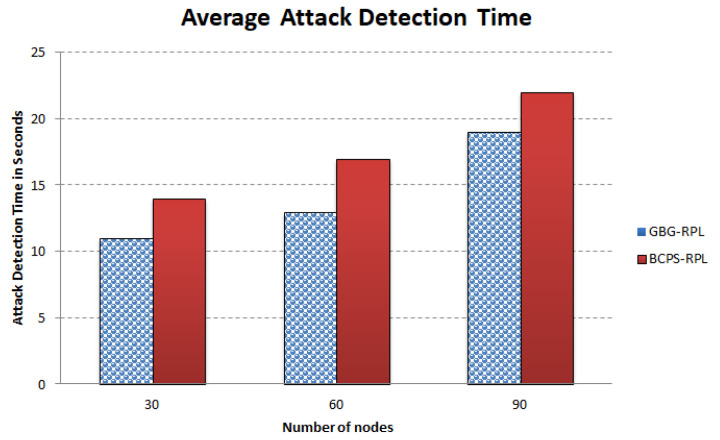
Attack-detection time.

In order to enable security experts to promptly take suitable measures in reaction to potential threats, it is desirable to have a low attack-detection time. Figure 12 shows how the number of nodes changed over time for the GBG-RPL and BCPS-RPL methods.

The potential of an approach to detect the existence of a node exhibiting abnormal behavior within the CPS network determines how long it takes to detect an attack. The GBG-RPL has a lower attack-detection time throughout the simulation time compared to BCPS-RPL, which can be attributed to three factors. Firstly, it is due to the careful selection of the trust parameters and configuration of the trickle timer. Secondly, the GBG-RPL performs all trust-related calculations in the fog server, which is a powerful computing device and reduces the processing time of trust calculations. Thirdly, the utilization of the Gini index improved the accuracy of the detection technique, as it provides granular-level details of defining a threshold to categorize behavior as normal or malicious. In contrast, in the case of the BCPS-RPL, numerous tasks, including trust-related processing, are being performed by the resource-constrained nodes, which take more time to identify a node behaving as abnormal. Furthermore, using packet drop as the only metric reduces the accuracy of the technique to detect abnormal behavior. Therefore, the overall attack-detection time of the GBG-RPL is better, which makes the attack-detection rate higher and enhances the reliability and efficiency of the CPS network.

### 10.7. Message Overhead

**Figure 13:**
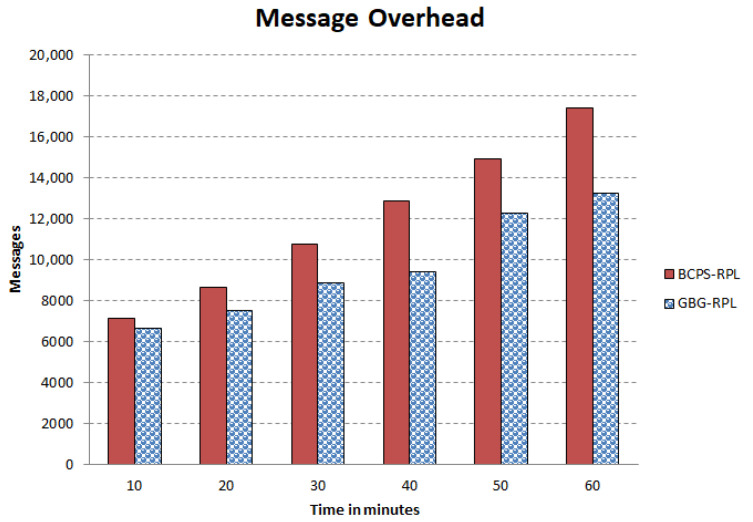
Message overhead.

Message overhead refers to the additional data or information that is transmitted along with the actual payload or user data during communication between nodes or systems in a network. Figure 13 shows the evolution of the message overhead for the BCPS-RPL and GBG-RPL processes.

Figure 13 illustrates the rising trend in the message overhead over time for both the BCPS-RPL mechanism and the GBG-RPL mechanism. The GBG-RPL mechanism consistently has a lower messaging overhead than the BCPS-RPL mechanism at each time interval, indicating a lighter communication load and less network congestion overall. Initially, both techniques show a similar message overhead as the network is being initialized and the trust calculations are being undertaken. However, after 20 min, the GBG-RPL technique considerably reduces the message overhead due to the fact that only trust parameters are being forwarded by the device layer and trust calculations are being carried out at the fog layer. Furthermore, the improved attack-detection time/rate contributed to the reduction in message overhead due to less frequent retransmissions of data/control packets.

### 10.8. Discussion

The successful implementation of the GBG-RPL framework hinges upon the collaborative efforts of diverse stakeholders. From healthcare data providers ensuring data quality to healthcare practitioners, each role is integral [104]. Data scientists optimize algorithms, auditors ensure security, and regulators monitor compliance [103]. Frontline healthcare providers utilize insights for enhanced care, and patient feedback contributes to ongoing refinement. This coordinated effort underscores the significance of each stakeholder in realizing the potential of the GBG-RPL framework in revolutionizing smart healthcare systems. An overview of the roles and responsibilities of the various stakeholders in a smart healthcare CPS is tabulated in Table 9.

In a smart healthcare system utilizing a Gini index-based trust framework, scalability requirements are crucial to ensure that the system can handle an increasing number of nodes, devices, and data transactions. The brief description of the scalability requirements is described below:

1. Node Growth: As the smart healthcare system expands, more medical devices, wearables, and IoT-enabled devices are added, increasing the number of nodes in the network [105]. The proposed framework will accommodate the growing number of nodes without significant degradation in performance due to its distributed nature [106].
2. Data Volume: With the proliferation of healthcare data generated by various devices, the system experiences an increase in the volume of data transactions [107]. The proposed framework efficiently handles the increased data volume, ensuring timely trust assessments without introducing delays due to the blockchain [108].
3. Real-Time Monitoring: The smart healthcare system requires real-time monitoring of patient data, vital signs, and device interactions for prompt decision making [109]. The proposed framework is capable of performing real-time trust assessments, adapting to dynamic changes in the network, and providing timely feedback as the fog layer is closer to the end-user devices.
4. Geographic Expansion: The smart healthcare system extends its services to new geographic locations, leading to a geographically distributed network [100]. The proposed framework is designed to support geographic expansion, considering potential latency issues and ensuring consistent trust management across distributed nodes.

Moreover, it is also important to define resource-management strategies. By implementing these strategies, the GBG-RPL framework ensures efficient and reliable trust management in the evolving landscape of smart healthcare systems. A brief description of the resource management strategies for implementing the GBG-RPL framework in smart healthcare CPSs is described below:

1. The proposed framework implements a distributed architecture for the Gini index where trust calculations and data processing are distributed across multiple fog nodes [110]. The proposed framework distributes the computational load, improving the overall system performance while enhancing the fault tolerance and resilience.
2. The proposed framework uses a layered architecture with a fog layer and a device layer [111]. The proposed framework prevents resource bottlenecks since all the trust calculation, analysis, and data storage are shifted to the fog layer, and the network underneath works efficiently.

In addition, securing the communications within the smart healthcare CPS is a paramount concern, necessitating the deployment of a robust set of security protocols [112]. In this complex ecosystem where the seamless exchange of sensitive healthcare data is pivotal, various protocols come into play to ensure the integrity, confidentiality, and authenticity of information during transit. The various security protocols for smart healthcare CPSs are described in Table 10.

The integration of the GBG-RPL framework into existing smart healthcare CPSs requires a systematic approach to ensure compatibility, functionality, and minimal disruption [119]. This integration involves careful planning, customization, and validation to ensure a seamless and effective deployment [4]. A step-by-step approach would help to navigate the complexities of integration, providing a solid foundation for enhanced trust management in healthcare data within the CPS [120]. A logical sequence of step-by-step approaches to the integration of GBG-RPL into smart healthcare CPSs is given in Table 11.

This research focused on the prevention of blackhole and greyhole attacks in smart healthcare CPSs using the Gini index and blockchain. The proposed mechanism consisted of two layers: the device layer and the fog layer. The Gini index and blockchain were implemented in the fog layer, where all trust-related calculations were performed. The model was implemented on the Cooja simulator. Several performance and efficiency parameters were evaluated in order to assess the proposed mechanism. Included in these parameters were the attack-detection rate, average residual energy, packet loss ratio, energy consumption, number of nodes, and end-to-end delay. Both the GBG-RPL and BCPS-RPL mechanisms demonstrated a consistent increase in attack-detection rate over time. Nevertheless, the GBG-RPL mechanism consistently displayed a greater attack-detection rate, indicating its effectiveness in detecting and mitigating blackhole and greyhole attacks.

Mean residual energy quantified the CPS energy levels over time. Both mechanisms’ energy consumption decreased over time, with the GBG-RPL mechanism consistently consuming less energy than the BCPS-RPL mechanism. This demonstrates the energy effectiveness of the GBG-RPL mechanism to maintain the CPS’s energy resources. The packet loss ratio parameter indicated the proportion of failed transmissions within the CPS network. The ratio of lost packets decreased over time for both mechanisms. However, the GBG-RPL mechanism consistently demonstrated a lower packet loss ratio than the BCPS-RPL mechanism, demonstrating its effectiveness in ensuring reliable data transmission within the system. Energy consumption was measured to evaluate the efficiency with which the mechanisms utilized energy resources. Both mechanisms’ energy consumption decreased over time, indicating improved energy efficiency in the CPS. The GBG-RPL mechanism consistently consumed less energy than the BCPS-RPL mechanism at each time interval, demonstrating its effectiveness in optimizing energy consumption.

The parameter representing the number of nodes quantified the network’s evolution over time. For both mechanisms, the number of nodes increased, with the GBG-RPL mechanism consistently having more nodes than the BCPS-RPL mechanism. This indicates that the GBG-RPL mechanism is scalable and capable of supporting a greater number of CPS network nodes. The end-to-end delay parameter measured the latency in message transmission within the CPS network based on the number of nodes. Both mechanisms’ end-to-end latencies increased as the number of nodes increased. The BCPS-RPL mechanism displayed marginally longer end-to-end delays than the GBG-RPL mechanism, indicating possible differences in their respective delay-management performance. The effectiveness of the GBG-RPL mechanism in preventing blackhole and greyhole attacks, optimizing energy consumption, minimizing packet loss, and ensuring reliable communication in the CPS network was demonstrated by the evaluation of the proposed mechanism using various parameters. The BCPS-RPL mechanism demonstrated scalability when coping with a larger number of nodes. This research provides essential insights for securing and optimizing CPS networks against malicious attacks and ensuring their effective operation.

Several performance metrics, including reliability, energy efficiency, security, scalability, and responsiveness, demonstrate that the GBG-RPL method outperforms the BCPS-RPL method, as shown in Table 12. In the table, the percentage of improvement was determined by averaging all values. However, in addition to the efficient attack detection, the proposed framework has some limitations. The initial assumption of network nodes’ trustworthiness may not align with the dynamic nature of evolving cybersecurity threats, and the static trust assumption could pose challenges in dynamically changing healthcare environments. Additionally, the assumption of a secure communication channel may not fully account for emerging cybersecurity threats. Moreover, the assumption that the attacker node is not intelligent may not hold against sophisticated adversaries, potentially compromising the framework’s effectiveness. Recognizing these limitations, real-world implementations must consider continuous evaluation, adaptation, and robust countermeasures to address potential vulnerabilities and ensure the framework’s resilience in dynamic healthcare cybersecurity landscapes.

## 11. Conclusions

This research undertook an in-depth study of the related work to identify the research gaps in the existing trust-based techniques to detect and mitigate blackhole and greyhole attacks in a CPS. The study considered the utilization of the Gini index and blockchain technology as an opportunity to enhance the security of CPS networks further. The Gini index can improve the accuracy of attack detection in trust-based schemes, whereas blockchain enhances the immutability of the trust database. The proposed framework calculates trust scores based on carefully selected significant trust parameters, including the packet drop ratio, energy consumption, end-to-end delay, and latency. The blockchain technology has been implemented as a database in the form of a smart contract to make the global trust list immutable. The trust calculation and implementation of smart contracts in the fog layer have improved the detection and mitigation of attacks, as observed during the simulations.

This research work proposes a thorough strategy for preventing blackhole and greyhole attacks in smart healthcare CPSs using the Gini index and blockchain. The suggested approach shows its usefulness in a range of CPS security and performance characteristics when implemented in the device layer and fog layer. When comparing the GBG-RPL mechanism to the BCPS-RPL mechanism, evaluations of several characteristics, including the attack-detection rate, average residual energy, packet loss ratio, energy consumption, number of nodes, and end-to-end delay, provide insightful information. In terms of attack-detection rate/time, energy efficiency, packet loss ratio, and transmission efficiency, the GBG-RPL method produces favorable outcomes. To validate and improve the suggested technique for real-world CPS deployments, nevertheless, more analysis and application in the field are needed. These research findings support ongoing efforts to increase the security and resistance of CPSs to complex attacks.

## Figures and Tables

**Table 1:** Related work on trust-based attack detection.

Reference	Technique	Attack	Centralized	Network Life	Scalability	Computation OH	Message OH	Energy OH
[17]	Trust	Internal	×	Medium	✓	Low	High	High
[32]	Trust	Sybil	✓	Low	×	High	High	High
[35]	Trust	Greyhole	✓	Low	×	High	High	High
[36]	Trust	Blackhole	✓	Low	×	High	High	High
[37]	Trust	Multiple	✓	Low	×	High	High	High
[43]	Trust	Multiple	✓	Low	×	High	High	High
[46]	Trust	Blackhole	✓	Low	×	Low	High	High
[47]	Trust	Blackhole	×	Low	×	High	High	High
[49]	Trust	Multiple	✓	Low	×	Low	High	High
[54]	Trust	Blackhole	✓	Low	×	High	High	High
[57]	Trust	Multiple	✓	Low	×	Low	High	High
[58]	Trust	Multiple	✓	Low	×	High	High	High
[59]	Trust	Multiple	✓	Low	×	High	High	High
[60]	Trust	Multiple	✓	Low	×	High	High	High
[61]	Trust	Multiple	×	Low	×	High	High	High

**Table 2:** Related research on blockchain.

Reference	Year	Pros	Cons	Practicality	Real-Time Applications
[17]	2020	Leverages blockchain for secure and tamper-proof storage of trust information.	Need for further validation and evaluation in real-world IoT scenarios.	✓	✓
[60]	2021	Uses data-driven approaches to detect and mitigate attacks.	Need for practical implementation and evaluation in real-world IoT deployments.	✓	✓
[61]	2021	Explores security concerns in fog computing and potential solutions using blockchain.	Lack of in-depth analysis and specific implementation details for blockchain solutions in fog computing.	✓	✓
[62]	2022	Explores the applications, benefits, and challenges of blockchain.	Some security aspects discussed may become outdated over time.	×	×
[63]	2023	Analyzes the challenges and opportunities of integrating blockchain into trust-management systems.	Fast-evolving nature of blockchain and IoT technologies may require frequent updates.	✓	✓
[64]	2022	Identifies the benefits, challenges, and potential applications of integrating blockchain with cloud computing.	Findings may become outdated due to the rapidly evolving nature of blockchain technology.	×	×
[65]	2022	Presents an overview of blockchain-based security solutions for IoT.	Lack of empirical evaluations and case studies to demonstrate the practical effectiveness of reviewed approaches.	×	×

**Table 3:** List of symbols used in mathematical notations.

Symbol	Meaning
*I*	Gini index (inequality of flow distribution in CPS network)
$a_{i}$	Proportion of flow *i* in the network
DR	Drop rate (the rate at which network packets are dropped or rejected)
*L*	Latency (delay experienced by packets during transmission)
*T*	Throughput (success rate of data transmission across the network)
$G_{m}$	Gini index value of node *m*
$P_{m}$	Packet loss rate of node *m*
$L_{m}$	Latency of node *m*
$Th_{m}$	Throughput of node *m*
*G*	Set of Gini index values for all nodes in the CPS network
Average(G)	Average Gini index value of all nodes in the CPS network
*N*	Set of entities in the system
Trust(N)	Trust values assigned to each entity in *N*
*D*	Set of dropped packets for each entity in *N*
*E*	Set of energy consumption values for each entity in *N*
*O*	Set of message overhead values for each entity in *N*
$G_{m}\left(T\right)$	Gini index value of node *m* at time *T*
$P_{m}\left(T\right)$	Packet loss rate of node *m* at time *T*
$L_{m}\left(T\right)$	Latency of node *m* at time *T*
$Th_{m}\left(T\right)$	Throughput of node *m* at time *T*
θ	Threshold value for node *m*
$\bar{G}$	Average value for the Gini index

**Table 4:** Description of parameters for direct trust.

Parameter	Description
PDR	Packet Drop Rate
EC	Energy Consumption
EED	End-to-End Delay
L	Latency

**Table 5:** Deployment models for proposed framework in smart healthcare CPS.

Model Type	Advantages	Disadvantages
Centralized [83]	Simplicity: Straightforward and easy to implement, especially for smaller-scale smart healthcare CPS. Control: Allows for easier management and coordination of security measures.	Single point of failure (SPOF): The centralized entity becomes an SPOF. Scalability issues: Challenges in scaling up for larger and more complex healthcare systems.
Hybrid [84]	Combines centralization and decentralization: Offers a balance between control and resilience. Scalability: More scalable than a purely centralized approach.	Complexity: Introduces complexity due to integration between centralized and decentralized components.
Fully Decentralized [85]	Resilience: More resilient against SPOF. Security: Improved security due to the absence of a central authority.	Complexity: Complex to implement and manage. Scalability challenges: Challenges, especially in large-scale smart healthcare CPS.
Fog Computing [86]	Reduced latency: Reduces latency and improves real-time decision making. Enhanced privacy: Reduces the need to transmit sensitive information across the network.	Consistency challenges: Ensuring consistent trust assessments across edge devices may require additional coordination mechanisms.
Cloud-Based [87]	Scalability: Allows for scalability by leveraging cloud resources. Resource management: Can better manage computational resources.	Dependency on cloud service providers (CSP): Reliance on external CSPs introduces a dependency. Security concerns: Security concerns due to the centralization of data.
Mesh Network [88]	Redundancy: Provides redundancy and resilience. Adaptability: Well-suited for dynamic healthcare environments.	Complex routing: Challenges in ensuring timely and efficient communication for trust parameter exchange. Resource consumption: Consumes more energy and resources compared to other models.

**Table 6:** Trust rating for CPS nodes.

Trust Value	Trust Status
0.7–1	Poor Trust
0.5–0.6	Less Fair Trust
0.2–0.4	Fair Trust
0.0–0.2	Good Trust

**Table 7:** Experimental setup.

Simulation Parameters	Value
OS/Platform	Linux
Simulation Software	Cooja 3.0
Nodes Used	30–90
Simulated Attacks	Blackhole and Greyhole
Attacker to Normal Node Ratio	1:10
Receive Ratio	30–100%
Transmit Ratio	100%
Transmission Range	50 m
Range of Interference	50 m
Protocol Used for Routing	RPL
Routine Trust Calculation	10 msec
Trickle Timer	5 msec
Initial Node Energy	100 J
Tx Energy	0.0010875 mJ/bit
Rx Energy	0.0009 mJ/bit
Standby Energy	0.708 mJ/s
Used Networking protocol	Internet Protocol-based
Time for Simulation	60 min
Reference Technique	BCPS-RPL [31]
Proposed Technique	GBG-RPL

**Table 8:** Node types and roles.

Node Type	Assigned Role
Full Node	Fog servers Has multiple functions Registers and maintains records of devices Calculates trust values Reviews, generates, and distributes global trust list Stores and maintains trust values in the blockchain
Root Node	Acts as a link between the device layer and fog layer Receives trust parameters from lower nodes Distributes updated trust list to lower nodes for the elimination of malicious nodes
Resource-Constrained Node	Resource-constrained device Performs only assigned tasks

**Table 9:** Roles and responsibilities in smart healthcare CPS.

Role	Responsibility
Healthcare Data Providers (Hospitals, Clinics, Laboratories)	Ensure data quality, integrity, and compliance with health regulations.
Smart Contract Developers	Design, develop, and maintain the GBG-RPL framework’s smart contracts and ensure the security and scalability of the blockchain infrastructure.
Data Scientists and Analysts	Optimize algorithms for the GBG-RPL framework and generate reports and visualizations for healthcare stakeholders.
Smart Contract Auditors	Review and audit the smart contracts of the GBG-RPL framework to identify and rectify security vulnerabilities.
Healthcare Regulators	Monitor the implementation of the GBG-RPL framework to ensure compliance with healthcare regulations and data protection laws.
Healthcare Providers (Doctors, Nurses, and Caregivers)	Use the insights from the GBG-RPL framework to improve patient care.
Patients and Healthcare Consumers	Provide feedback on the usability and relevance of the index.

**Table 10:** Communication security protocols for smart healthcare CPS.

Type	Description	Security Aspect
Access Controls and Identity Management [69]	Ensure communication access to authorized entities within the CPS.	Prevent unauthorized access and establish trusted communication channels.
TLS and SSL [113]	Provide encryption and authentication for end-to-end security of data in transit.	Protect against eavesdropping and MITM attacks.
MAC and Digital Signatures [114]	Verify the authenticity and integrity of transmitted messages.	Ensure that data remain unchanged and originate from a legitimate source.
VPNs [115]	Create encrypted tunnels between nodes.	Enhance privacy and security.
PKC [116]	Ensures that data remain confidential from the point of origin to the destination.	Minimize the risk of interception and unauthorized access.
SSH [117]	Encrypts communication sessions for remote access and command execution.	Adds an extra layer of protection against unauthorized access and data tampering.
Network Segmentation [118]	Divides the network into segments with restricted access.	Limits the impact of unauthorized access and reduces the attack surface.

**Table 11:** Integration of GBG-RPL framework into smart healthcare CPS.

Sequence of Integration	Purpose	Actions
Assess Existing Infrastructure	Understanding of the current architecture, components, and communication protocols of the smart healthcare CPS.	Conduct a thorough assessment of the existing infrastructure.
Identify Integration Points	Determine specific points within the smart healthcare CPS where the GBG-RPL framework would be integrated.	Identify areas such as trust-management modules and communication interfaces where the GBG-RPL can be integrated.
Define Data Exchange Protocols	Establish standardized protocols for the exchange of data between the GBG-RPL framework and existing components of the smart healthcare CPS.	Define communication standards, data formats, and protocols to ensure interoperability.
Adapt GBG-RPL to the Healthcare Domain	Tailor the GBG-RPL algorithms and parameters to suit the specific requirements and characteristics of healthcare.	Customize the GBG-RPL to handle healthcare-related trust metrics.
Ensure Security Measures	Address security considerations to protect healthcare data and maintain the integrity of trust assessments.	Implement hashing and layered architecture to safeguard data exchanged between the Gini index and other components.
Testing and Validation	Verify the integration’s functionality, performance, and security through comprehensive testing.	Conduct integration testing to validate compatibility with existing CPS components.
User Training and Adoption	Prepare healthcare professionals, administrators, and other users for the introduction of the proposed framework, ensuring they understand its role and benefits.	Provide training sessions, workshops, and documentation.
Test Deployment	Conduct a test deployment in a controlled environment.	Deploy the proposed framework on a limited scale, monitor its operation, and collect feedback.
Optimization and Full Deployment	Implement necessary optimizations based on feedback.	Make refinements to the integration.

**Table 12:** Performance comparison of GBG-RPL and BCPS-RPL.

Metric	GBG-RPL	BCPS-RPL	% Improvement
Packet Loss Ratio	0.375	0.404	7.18%
Residual Energy	0.635	0.559	11.97%
Energy Consumption	0.04768	0.0591	19.27%
Attack-Detection Rate	0.77	0.688	10.65%
Avg. Attack-Detection Time (30 nodes)	14.33	17.67	18.88%
Message Overhead	9435	12050	21.65%
End-to-End Delay (30 nodes)	0.907	1.267	28.34%

## Data Availability

Data sharing not applicable.

## Abbreviations

List of abbreviations:

**Abbreviation**	**Meaning**
AI	Artificial Intelligence
BCPS-RPL	Blackhole detection in RPL-based CPS
CPS	Cyber-Physical System
DAO	Destination Advertisement Object
DODAG	Destination-Oriented Directed Acyclic Graph
DL	Deep Learning
DoS	Denial of Service
GBG-RPL	Gini-index and blockchain-based Blackhole/Greyhole RPL
Greyhole	Greyhole Attack
Gini	Gini Logic
IDS	Intrusion-Detection System
IoT	Internet of Things
ITS	Intelligent Transportation System
LLN	Low-Power Lossy Networks
LBR	LLN Border Router
MAC	Message Authentication Code
MITM	Man-in-the-Middle
ML	Machine Learning
mj	MilliJoules
OS	Operating System
PKC	Public Key Cryptography
RAM	Random Access Memory
BRGTL	Blockchain-based Revised Global Trust List
RPL	Routing Protocol for Low-power Lossy Networks
SHA-256	Secure Hash Algorithm 256
SSH	Secure Shell
SN	Sensor Nodes
SPOF	Single Point of Failure
TLS	Transport Layer Security
VPN	Virtual Private Network
WSN	Wireless Sensor Network
ERT	Electrical Resistivity Tomography Algorithm
6LoWPAN	IPv6 LLN Private Access Networks
